# Supplementary material for: Development of a short-form Swedish version of the Montreal Cognitive Assessment (s-MoCA-SWE): protocol for a cross-sectional study
Source: BMJ Open. 2021 May 3;11(5):e049035. doi: 10.1136/bmjopen-2021-049035 (PMC8098968; doi:10.1136/bmjopen-2021-049035)
Supplement: Supplementary data [file bmjopen-2021-049035supp001.pdf]

Supplementary File

Development of a short-form Swedish version of the Montreal Cognitive Assessment (s-MoCA-SWE): Protocol for a cross-sectional study

Tamar Abzhandadze[1,2], Erik Lundström[3], Dongni Buvarp[1], Marie Eriksson[4], Terence J. Quinn [5], Katharina S. Sunnerhagen[1,6].

[1]Institute of Neuroscience and Physiology, The Sahlgrenska Academy, University of Gothenburg, Gothenburg, Sweden.

[2]Department of Occupational Therapy and Physiotherapy, Sahlgrenska University Hospital, Gothenburg, Sweden.

[3]Department of Neuroscience, Neurology, Uppsala University, Akademiska Sjukhuset, Uppsala, Sweden.

[4]Department of Statistics, USBE, Umeå University, Umeå, Sweden.

[5]Institute of Cardiovascular and Medical Sciences, University of Glasgow, Glasgow, UK.

[6] Neurocare, Sahlgrenska University Hospital, Gothenburg, Sweden.

Correspondence to:

Tamar Abzhandadze

Institute of Neuroscience and Physiology

Rehabilitation Medicine, University of Gothenburg

Per Dubbsgatan 14, fl. 3

413 45 Gothenburg, Sweden

E-mail: tamar.abzhandadze@gu.se, tamar.abzhandadze@vgregion.se

Supplementary Table 1. Description of variables and their categories respective data set and in the merged dataset.

**Väststroke**	**Väststroke: variable categories and coding**	**EFFECTS dataset**	**EFFECTS: variable categories and coding**	**Merged dataset: variable names and coding**
				Cohorts, binary:<br>1 = Väststroke<br>2 = EFFECTS
Sex	Binary:<br>0 = female<br>1 = male	Pat_Gender	F = female<br>M = male	Sex, binary:<br>1 = female<br>2 = male
Age	Scale: range	AGE	Numeric	Age: numeric
Living arrangement/living alone prior to stroke	Binary:<br>1 = no<br>2 = yes	pat_Living_Arrangements	A = living alone<br>B = institutional living<br>C = other living arrangement<br>D = living with someone else	Living arrangement prior to stroke, binary:<br>1 = lived alone (Väststroke = 2, EFFECTS = A)<br>2 = did not live alone (Väststroke = 1, EFFECTS ≥B)
Needing help/dependent prior to stroke	Binary:<br>1 = no<br>2 = yes	Stroke daily	N = no<br>Y = yes<br><br>*Note: "N" means that the patient did not require assistance with ADLs prior to stroke.*	Needs assistance with ADL before stroke, binary:<br>1 = no<br>2 = yes
Diabetes	Binary:<br>0 = no<br>1 = yes	DIABETES	N = no<br>Y = yes<br>U = unknown	Diabetes, nominal:<br>0 = no<br>1 = yes<br>2 = unknown

TIA prior to stroke	Binary:<br>0 = no<br>1 = yes	PREV_Stroke_TIA<br><br>*Note: In EFFECTS, previous TIA and previous ischemic stroke are not distinguished.*	N = no<br>Y = yes<br>U = unknown	Previous TIA or ischemic stroke, nominal:<br>0 = no<br>1 = yes<br>2 = unknown
Stroke diagnosis	Nominal, 3 levels:<br>1 = I61<br>2 = I63<br>3 = I64	Diagnosis	1 = ischemic stroke<br>2 = intracerebral haemorrhage<br>3 = non-stroke	Stroke diagnosis, nominal:<br>1 = I63<br>2 = I61<br>3 = I64
Reperfusion	1 = both IVT and endovascular thrombectomy<br>2 = endovascular thrombectomy only<br>3 = IVT only	Thrombolysis_Trombectomy	1 = both IVT and endovascular thrombectomy<br>2 = endovascular thrombectomy only<br>3 = IVT only	Reperfusion treatment, nominal:<br>1 = both IVT and endovascular thrombectomy<br>2 = endovascular thrombectomy only<br>3 = IVT only
NIHSS awareness	Ordinal: range 0–3	Stroke_NIHSS_1a	0 = alert<br>1 = not alert, but arousable with minimal stimulation<br>2 = not alert, requires repeated stimulation<br>3 = coma	NIHSS 1a, awareness, ordinal:<br>0 = alert<br>1 = not alert, but arousable with minimal stimulation<br>2 = not alert, requires repeated stimulation<br>3 = coma
NIHSS orientation	Ordinal: range 0–2	Stroke_NIHSS_1b	0 = answers both correctly<br>1 = answers one correctly<br>2 = answers both incorrectly	NIHSS 1b, orientation, ordinal:<br>0 = answers both correctly<br>1 = answers one correctly<br>2 = answers both incorrectly

NIHSS understanding	Ordinal: range 0–2	Stroke_NIHSS_1c	0 = obeys both correctly<br>1 = obeys one correctly<br>2 = obeys both incorrectly	NIHSS 1c, understanding, ordinal:<br>0 = obeys both correctly<br>1 = obeys one correctly<br>2 = obeys both incorrectly
NIHSS eye movement	Ordinal: range 0–2	Stroke_NIHSS_2	0 = normal<br>1 = partial gaze palsy<br>2 = total gaze paresis	NIHSS 2, eye movement, ordinal:<br>0 = normal<br>1 = partial gaze palsy<br>2 = total gaze paresis
NIHSS visual field	Ordinal: range 0–3	Stroke_NIHSS_3	0 = no visual field loss<br>1 = partial hemianopia<br>2 = complete hemianopia<br>3 =bilateral hemianopia	NIHSS 3, visual field, ordinal:<br>0 = no visual field loss<br>1 = partial hemianopia<br>2 = complete hemianopia<br>3 = bilateral hemianopia
NIHSS facial palsy	Ordinal: range 0–3	Stroke_NIHSS_4	0 = normal symmetrical movement<br>1 = minor paralysis<br>2 = partial paralysis<br>3 = complete paralysis	NIHSS 4, facial palsy, ordinal:<br>0 = normal symmetrical movement<br>1 = minor paralysis<br>2 = partial paralysis<br>3 = complete paralysis
NIHSS motor arm: right	Ordinal: range 0–4	Stroke_NIHSS_7	0 = normal<br>1 = drift<br>2 = some effort against gravity<br>3 = no effort against gravity<br>4 = no effort against gravity	NIHSS 5, right motor arm, ordinal:<br>0 = normal<br>1 = drift<br>2 = some effort against gravity<br>3 = no effort against gravity<br>4 = no effort against gravity

NIHSS motor arm: left	Ordinal: range 0–4	Stroke_NIHSS_8	0 = normal<br>1 = drift<br>2 = some effort against gravity<br>3 = no effort against gravity<br>4 = no effort against gravity	NIHSS 6, left motor arm, ordinal:<br>0 = normal<br>1 = drift<br>2 = some effort against gravity<br>3 = no effort against gravity<br>4 = no effort against gravity
NIHSS motor leg: right	Ordinal: range 0–4	Stroke_NIHSS_5	0 = normal<br>1 = drift<br>2 = some effort against gravity<br>3 = no effort against gravity<br>4 = no effort against gravity	NIHSS 7, right motor leg, ordinal:<br>0 = normal<br>1 = drift<br>2 = some effort against gravity<br>3 = no effort against gravity<br>4 = no effort against gravity
NIHSS motor leg: left	Ordinal: range 0–4	Stroke_NIHSS_6	0 = normal<br>1 = drift<br>2 = some effort against gravity<br>3 = no effort against gravity<br>4 = no effort against gravity	NIHSS 8, left motor leg, ordinal:<br>0 = normal<br>1 = drift<br>2 = some effort against gravity<br>3 = no effort against gravity<br>4 = no effort against gravity
NIHSS ataxia	Ordinal: range 0–2	Stroke_NIHSS_9	0 = no ataxia<br>1 = present in one limb<br>2 = present in two limbs	NIHSS 9, ataxia, ordinal:<br>0 = no ataxia<br>1 = present in one limb<br>2 = present in two limbs
NIHSS sensory	Ordinal: range 0–2	Stroke_NIHSS_10	0 = normal<br>1 = mild-to-moderate sensory loss<br>2 = severe or total sensory loss	NIHSS 10, sensory, ordinal:<br>0 = normal<br>1 = mild-to-moderate sensory loss<br>2 = severe or total sensory loss

NIHSS language	Ordinal: range 0–3	Stroke_NIHSS_11	0 = no aphasia<br>1 = mild-to-moderate aphasia<br>2 = severe aphasia<br>3 = mute	NIHSS 11, language, ordinal:<br>0 = no aphasia<br>1 = mild-to-moderate aphasia<br>2 = severe aphasia<br>3 = mute
NIHSS dysarthria	Ordinal: range 0–2	Stroke_NIHSS_12	0 = normal<br>1 = mild-to-moderate dysarthria<br>2 = severe dysarthria<br>3 = untestable	NIHSS 12, dysarthria, ordinal:<br>0 = normal<br>1 = mild-to-moderate dysarthria<br>2 = severe dysarthria<br>3 = untestable
NIHSS neglect	Ordinal: range 0–2	Stroke_NIHSS_13	0 = normal<br>1 = inattention to one modality<br>2 = severe hemi-inattention	NIHSS 13, neglect, ordinal:<br>0 = normal<br>1 = inattention to one modality<br>2 = severe hemi-inattention
NIHSS total score	Ordinal: range 0–36	NIHSS	Ordinal: range 0–26	NIHSS total score, ordinal: range 0–36
MoCA visuospatial/executive functions: 1A + cube + clock	Ordinal: range 0–5	MOCA_Base_Viso_exe	Ordinal: range 0–5	MoCA visuospatial/executive functions: trial + cube + clock, ordinal: range 0–5
MoCA task: naming	Ordinal: range 0–3	MOCA_Base_Name	Ordinal: range 0–3	MoCA: naming, ordinal: range 0–3
MoCA attention task: numbers forward and backward	Ordinal: range 0–2	MOCA_Base_Att_fwd_back	Ordinal: range 0–2	MoCA: digit span, ordinal: range 0–2
MoCA attention task: knock on A	Nominal:<br>0 = no point<br>1 = 1 point	MOCA_Base_Att_knock	Nominal:<br>0 = no point<br>1 = 1 point	MoCA: tap on A, binary:<br>0 = no point<br>1 = 1 point
MoCA attention task: subtraction 100 - 7	Ordinal: range 0–3	MOCA_Base_Att_subtract	Ordinal: range 0–3	MoCA: serial 7, ordinal: range 0–3

MoCA language task: sentence repetition, 2	Ordinal: range 0–2	MOCA_Base_Lang_rep	Ordinal: range 0–2	MoCA: repetition, ordinal: range 0–2
MoCA language task: fluency, F word	Nominal:<br>0 = no point<br>1 = 1 point	MOCA_Base_Fluency	Nominal:<br>0 = no point<br>1 = 1 point	MoCA: fluency, binary:<br>0 = no point<br>1 = 1 point
MoCA abstraction task: categorizing of two tasks	Ordinal: range 0–2	MOCA_Base_Abstraction	Ordinal: range 0–2	MoCA: abstraction, ordinal: range 0–2
MoCA memory task: recall of five words	Ordinal: range 0–5	MOCA_Base_Delay	Ordinal: range 0–5	MoCA: delayed recall, ordinal: range 0–5
MoCA orientation task: six questions	Ordinal: range 0–6	MOCA_Base_Orientation	Ordinal: range 0–6	MoCA: orientation, ordinal: range 0–6
MoCA total score	Ordinal: range 1–30	MOCA_Base_Total	Ordinal: range 1–30	MoCA total score, ordinal: range 1–30
MoCA school ≤12 years, +1 point	Binary:<br>0 = no<br>1 = yes, +1 point	MOCA_Education	0 = no<br>1 = yes, +1 point	MoCA education, binary:<br>0 = no,<br>1 = yes, +1 point
				A telephone version of the MoCA, ordinal: range 0–22<br><br>Aggregated score of the MoCA items: delayed recall, digit span, list letters, serial 7, sentence repetition, fluency, abstraction, and orientation
				Short-form MoCA, ordinal: range 0–14<br><br>Aggregated score of the MoCA items: delayed recall, serial 7, and orientation

				NINDS-CSN–MoCA, ordinal: range 0–12<br><br>Aggregated score of the MoCA items: delayed recall, fluency, and orientation
Discharge destination*	Ordinal, 10 levels:<br>1 = own accommodation<br>2 = arranged accommodation<br>4 = another acute-care department<br>5 = geriatrics/rehabilitation<br>6 = deceased during treatment<br>7 = other acute-care department (e.g. the patient is resident in another country)<br>9 = not known<br>11 = still in hospital<br>12 = another stroke unit for aftercare<br>13 = health centre with emergency beds	Discharge residence	1 = own home<br>2 = relatives' home<br>3 = residential home<br>4 = long-term NHS home<br>6 = another hospital<br>7 = other	Discharge destination, ordinal:<br>1 = own or relatives' home (Väststroke 1, EFFECTS 1 and 2)<br>2 = residential home (Väststroke 2, EFFECTS 3 and 4)<br>3 = other hospital (Väststroke 4, 5, 11, and 12, EFFECTS 6)<br>4 = other (Väststroke 7, 9, and 13, EFFECTS 7)

Abbreviations: ADL, activity of daily living; IVT, intravenous thrombolysis; MoCA, Montreal Cognitive Assessment; NIHSS, National Institutes of Health Stroke Scale; NINDS-CSN, National Institute of Neurological Disorders and Stroke–Canadian Stroke Network; TIA, transient ischemic attack.

Supplementary Table 2. Patient baseline characteristics (template table)

	Categories	Total sample<br>N =	Väststroke<br>N =	EFFECTS<br>N =
Sex, n (%)	Male<br>Female			
Age, y, mean (SD)				
Living arrangement, n (%)	Lives alone<br>Living with someone else			
Education, 1 point for a maximum of 12 years, n (%)	Yes<br>No			
Assistance with ADLs prior to stroke, n (%)	No<br>Yes			
Having diabetes, n (%)	No<br>Yes<br>Unknown			
Previous TIA or ischemic stroke, n (%)	No<br>Yes<br>Unknown			
Stroke diagnosis, n (%)	I61<br>I63<br>I64			

Reperfusion treatment, n (%)	Both IVT and ET<br>ET only<br>IVT only			
NIHSS, median (min–max)				
MoCA, median (min–max)				
Discharge destination, n (%)	Own or relatives' home<br>Residential or long-term<br>NHS home<br>Other hospitals<br>Other			

Note: The sum may vary due to missing data.

Abbreviations: ADL, activity of daily living; IVT, intravenous thrombolysis; IQR, interquartile range; MoCA, Montreal Cognitive Assessment; NIHSS, National Institutes of Health Stroke Scale; NHS, National Health System; SD, standard deviation; ET, endovascular thrombectomy; TIA, transient ischemic attack.

Variables with missing values, n (%):

Supplementary Table 3. Description of cognitive functions (template table).

		Total sample<br>N =	Väststroke<br>N =	EFFECTS<br>N =
	Points	n (%)	n (%)	n (%)
Visuospatial/executive functions	0<br>1<br>2<br>3<br>4<br>5			
Naming three animals	0<br>1<br>2<br>3			
Digit span	0<br>1<br>2			
Tap on A	0<br>1			
Serial 7	0<br>1			

	2<br>3			
Repetition	0<br>1<br>2			
Fluency	0<br>1			
Abstraction	0<br>1<br>2			
Delayed recall	0<br>1<br>2<br>3<br>4<br>5			
Orientation	0<br>1<br>2<br>3<br>4			

	5<br>6			
MoCA score	Median (IQR)			
T-MoCA	Median (IQR)			
NINDS-CSN–MoCA	Median (IQR)			
SF-MoCA	Median (IQR)			

Abbreviations: IQR, interquartile range; MoCA, Montreal Cognitive Assessment (score range: 1–30); NINDS-CSN–MoCA, National Institute of Neurological Disorders and Stroke–Canadian Stroke Network MoCA (score range: 0–12); SF-MoCA, short-form MoCA (score range: 0–14); T-MoCA, a telephone version of the MoCA (score range: 0–22).

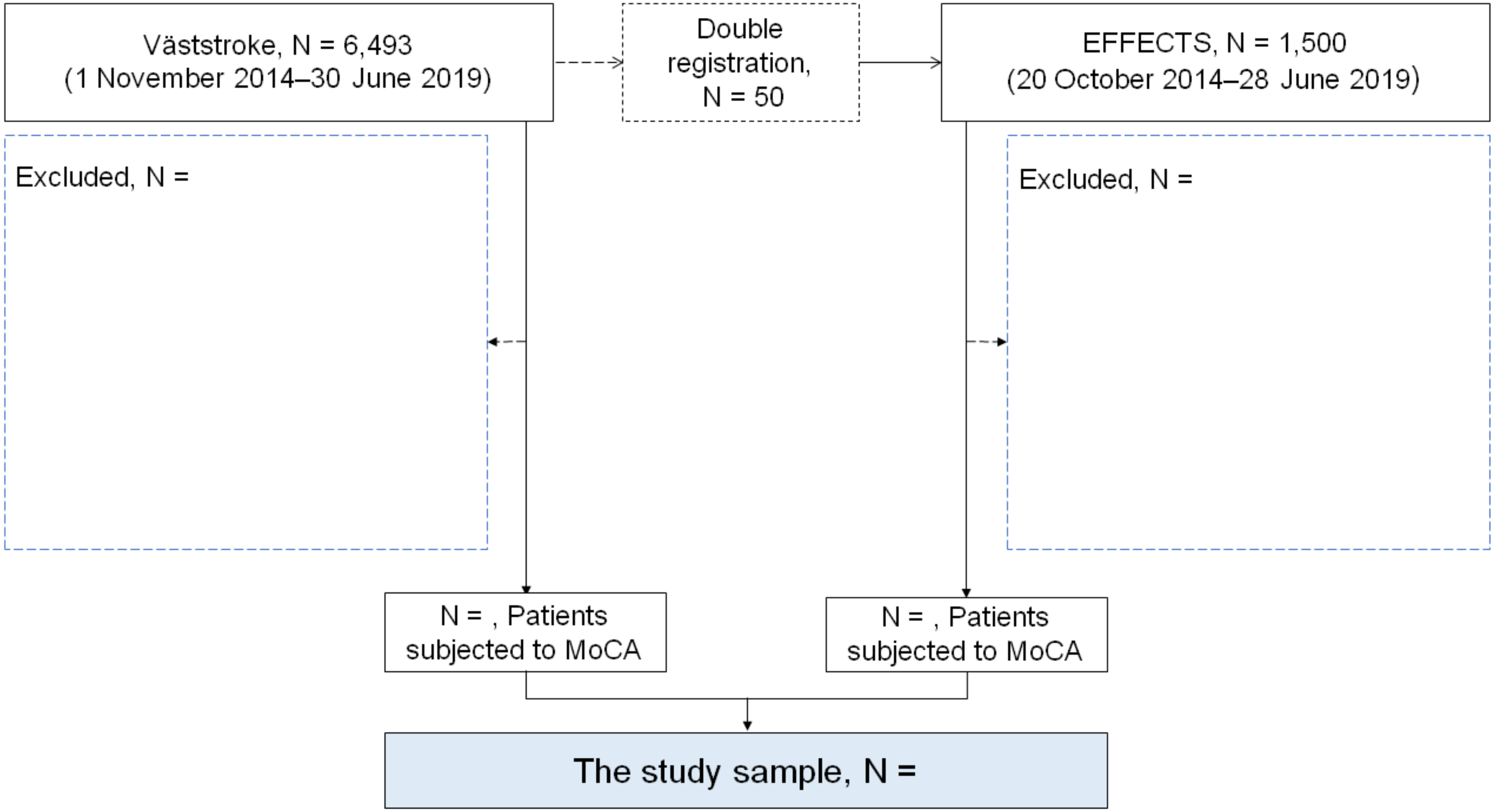

Supplementary Figure. Flowchart of patient selection (template diagram). Abbreviations: MoCA, the Montreal Cognitive Assessment; EFFECTS, **E**fficacy o**F** **F**luoxetine—a randomis**E**d **C**ontrolled **T**rial in **S**troke.
