# Supplementary material for: Development of a short-form Swedish version of the Montreal Cognitive Assessment (s-MoCA-SWE): protocol for a cross-sectional study
Source: BMJ Open. 2021 May 3;11(5):e049035. doi: 10.1136/bmjopen-2021-049035 (PMC8098968; doi:10.1136/bmjopen-2021-049035)
Supplement: Author's manuscript [file bmjopen-2021-049035.draft_revisions.pdf]

BMJ Open

BMJ Open is committed to open peer review. As part of this commitment we make the peer review history of every article we publish publicly available.

When an article is published we post the peer reviewers' comments and the authors' responses online. We also post the versions of the paper that were used during peer review. These are the versions that the peer review comments apply to.

The versions of the paper that follow are the versions that were submitted during the peer review process. They are not the versions of record or the final published versions. They should not be cited or distributed as the published version of this manuscript.

BMJ Open is an open access journal and the full, final, typeset and author-corrected version of record of the manuscript is available on our site with no access controls, subscription charges or pay-per-view fees (http://bmjopen.bmj.com).

If you have any questions on BMJ Open's open peer review process please email info.bmjopen@bmj.com

BMJ Open

Development of a short-form Swedish version of the Montreal Cognitive Assessment (s-MoCA-SWE): Protocol for a cross-sectional study

Journal:	*BMJ Open*
Manuscript ID	bmjopen-2021-049035
Article Type:	Protocol
Date Submitted by the Author:	14-Jan-2021
Complete List of Authors:	Abzhandadze, Tamar; University of Gothenburg Sahlgrenska Academy, Institute of Neuroscience and Physiology; Sahlgrenska University Hospital, Department of Occupational Therapy and Physiotherapy<br>Lundström, Erik; Akademiska Sjukhuset, Department of Neuroscience<br>Buvarp, Dongni; University of Gothenburg, Institute of Neuroscience and Physiology<br>Eriksson, Marie; Umeå University, Department of Statistics, USBE<br>Quinn, Terry; University of Glasgow, Cardiovascular and Medical Sciences<br>Sunnerhagen, Katharina; University of Gothenburg, Institute of Neuroscience and Physiology; Sahlgrenska University Hospital, Neurocare
Keywords:	STROKE MEDICINE, Stroke < NEUROLOGY, REHABILITATION MEDICINE

SCHOLARONE™
Manuscripts

I, the Submitting Author has the right to grant and does grant on behalf of all authors of the Work (as defined in the below author licence), an exclusive licence and/or a non-exclusive licence for contributions from authors who are: i) UK Crown employees; ii) where BMJ has agreed a CC-BY licence shall apply, and/or iii) in accordance with the terms applicable for US Federal Government officers or employees acting as part of their official duties; on a worldwide, perpetual, irrevocable, royalty-free basis to BMJ Publishing Group Ltd ("BMJ") its licensees and where the relevant Journal is co-owned by BMJ to the co-owners of the Journal, to publish the Work in this journal and any other BMJ products and to exploit all rights, as set out in our licence.

The Submitting Author accepts and understands that any supply made under these terms is made by BMJ to the Submitting Author unless you are acting as an employee on behalf of your employer or a postgraduate student of an affiliated institution which is paying any applicable article publishing charge ("APC") for Open Access articles. Where the Submitting Author wishes to make the Work available on an Open Access basis (and intends to pay the relevant APC), the terms of reuse of such Open Access shall be governed by a Creative Commons licence – details of these licences and which Creative Commons licence will apply to this Work are set out in our licence referred to above.

Other than as permitted in any relevant BMJ Author's Self Archiving Policies, I confirm this Work has not been accepted for publication elsewhere, is not being considered for publication elsewhere and does not duplicate material already published. I confirm all authors consent to publication of this Work and authorise the granting of this licence.

For peer review only

Development of a short-form Swedish version of the Montreal Cognitive Assessment (s-MoCA-SWE): Protocol for a cross-sectional study

Tamar Abzhandadze[1,2], Erik Lundström[3], Dongni Buvarp[1], Marie Eriksson[4], Terence J. Quinn [5], Katharina S. Sunnerhagen[1,6].

[1]Institute of Neuroscience and Physiology, The Sahlgrenska Academy, University of Gothenburg, Gothenburg, Sweden.

[2]Department of Occupational Therapy and Physiotherapy, Sahlgrenska University Hospital, Gothenburg, Sweden.

[3]Department of Neuroscience, Neurology, Uppsala University, Akademiska Sjukhuset, Uppsala, Sweden.

[4]Department of Statistics, USBE, Umeå University, Umeå, Sweden.

[5]Institute of Cardiovascular and Medical Sciences, University of Glasgow, Glasgow, UK.

[6] Neurocare, Sahlgrenska University Hospital, Gothenburg, Sweden.

Correspondence to:

Tamar Abzhandadze

Institute of Neuroscience and Physiology

Rehabilitation Medicine, University of Gothenburg

Per Dubbsgatan 14, fl. 3

413 45 Gothenburg, Sweden

E-mail: tamar.abzhandadze@gu.se, tamar.abzhandadze@vgregion.se

Word count, excluding title page, abstract, references, figures, and tables: 2,603

For peer review only - http://bmjopen.bmj.com/site/about/guidelines.xhtml

ABSTRACT

Introduction

Short forms of the Montreal Cognitive Assessment (MoCA) have allowed quick cognitive screening. However, none of the available short forms have been developed or validated in a Swedish sample of patients with stroke.

The aim is to develop a short-form Swedish version of the Montreal Cognitive Assessment (s-MoCA-SWE) in a sample of patients with acute and subacute stroke. The specific objectives are: (1) to identify a subgroup of MoCA items that have the potential to form the s-MoCA-SWE; (2) to determine the optimal cutoff value of s-MoCA-SWE for predicting cognitive impairment; (3) and to compare the psychometric properties of s-MoCA-SWE with those of previously developed MoCA short forms.

Methods and analysis

This is a cross-sectional study. The study sample will comprise patients from Väststroke, a local stroke registry from Gothenburg, Sweden and **E**fficacy o**F** **F**luoxetine—a randomis**E**d **C**ontrolled **T**rial in **S**troke (EFFECTS), a randomised controlled trial in Sweden. The s-MoCA-SWE will be developed by using exploratory factor analysis and the boosted regression tree algorithm. The cutoff value of s-MoCA-SWE for impaired cognition will be determined based on binary logistic regression analysis. The psychometric properties of s-MoCA-SWE will be compared with those of other MoCA short forms by using cross-tabulation and area under the receiving operating characteristic curve analyses.

Ethics and dissemination

The Väststroke study has received ethical approval from the Regional Ethical Review Board in Gothenburg (346–16) and the Swedish Ethical Review Authority (amendment 2019–04299). The handling of data generated within the framework of quality registers does not require written informed consent from patients. The EFFECTS study has received ethical approval from the Stockholm Ethics Committee (2013/1265-31/2 on 30/09/2013). All participants provided written consent. Results will be published in an international, peer-reviewed journal, presented at conferences, and communicated to clinical practitioners in local meetings and seminars.

For peer review only - http://bmjopen.bmj.com/site/about/guidelines.xhtml

Key words: cognitive screening, acute and subacute stroke, statistical analyses plan, development, validation.

For peer review only

For peer review only - http://bmjopen.bmj.com/site/about/guidelines.xhtml

STRENGTHS AND LIMITATIONS OF THIS STUDY

- A short form of the Swedish version of the Montreal Cognitive Assessment will be developed for the first time.
- The study will use a large sample size from different acute stroke and stroke rehabilitation units across Sweden.
- Data will be analysed using various statistical approaches.
- The retrospective analysis of registry data is one limitation of the study.
- A high number of non-random missing Montreal Cognitive Assessment values is expected.

For peer review only

INTRODUCTION

Cognitive impairment, a common sequela of stroke, has been defined as a new cognitive deficit that develops in the first 3 months after stroke and cannot be explained by other conditions or diseases.[1] Cognitive impairment is a consequence of complex interactions between age, education, and injury size and location.[2] The prevalence of cognitive impairment in the first 3 weeks after stroke can vary between 55% and 59% depending on the time of assessment and the assessment instruments.[3, 4] Previous studies have shown that cognitive impairment is associated with higher mortality and long-term disability.[5] Thus, early is important for planning individually tailored rehabilitation.

Comprehensive cognitive assessments in acute stroke units can be time- and resource-demanding for patients as well as for healthcare professionals. Therefore, the use of short screening tools for cognitive impairment is recommended.[6, 7] The Montreal Cognitive Assessment (MoCA) is frequently used to assess cognitive function.[7] The MoCA targets cognitive domains such as visuospatial ability, executive function, attention, concentration, working memory, language, short-term memory, and orientation.[8] It has a good sensitivity for detecting post-stroke cognitive impairment.[9, 10] To make cognitive screenings time effective, several short forms of the MoCA have been developed and tested in recent years, including a telephone version of the MoCA (T-MoCA), a short version of the MoCA that has been designed by the National Institute of Neurological Disorders and Stroke and the Canadian Stroke Network (NINDS-CSN, 5-minute protocol), and the short-form MoCA (SF-MoCA).[11-19] Different cutoffs for impaired cognition have been reported for the MoCA as well as its short forms.[11, 12, 15, 17-20] None of the available short forms have been developed or validated in a Swedish sample of patients with stroke.

Performance on cognitive tests after stroke can depend on premorbid cognitive reserves, which are associated with education, occupation, and socioeconomic situation.[21, 22] Items of the MoCA can function differently in various cultures; therefore, cultural validation of the MoCA has been recommended for generalisability.[23] Theoretically, this recommendation is accurate even when it comes to short forms of the MoCA. In order to have a short form of the MoCA used in a broader context as a time-effective screening instrument with good sensitivity for detecting cognitive impairment, it needs to be validated culturally within the target population.

For peer review only - http://bmjopen.bmj.com/site/about/guidelines.xhtml

Study aims

The aim of this study is to develop a short-form Swedish version of the Montreal Cognitive Assessment (s-MoCA-SWE) using data from Swedish stroke cohorts. The specific objectives are: (1) to identify a subgroup of MoCA items that have the potential to form the s-MoCA-SWE; (2) to determine the optimal cutoff value of s-MoCA-SWE for predicting cognitive impairment; and (3) to compare the psychometric properties of s-MoCA-SWE with those of T-MoCA, NINDS-CSN, and SF-MoCA.

METHODS AND ANALYSIS

Study design

This is a cross-sectional, exploratory study. Two datasets will be used: Väststroke, a local stroke registry from Gothenburg, Sweden and **E**fficacy o**F** **F**luoxetine—a randomis**E**d **C**ontrolled **T**rial in **S**troke (EFFECTS), a randomised controlled trial.[24]

The Väststroke register comprises data from three stroke units in Sahlgrenska University Hospital. The Väststroke registry was linked to the Riksstroke, a national quality register for stroke care in Sweden, via each patient's unique personal identification number.[25] The statisticians at Riksstroke merged the data. The Väststroke data from 1 November 2014 to 30 June 2019 were retrieved.

The EFFECTS was a randomized, double-blind, placebo-controlled clinical trial conducted in 35 stroke or rehabilitation units in Sweden.[24] The inclusion period was from 20 October 2014 to 28 June 2019.[24] Briefly, patients aged ≥ 18 years and diagnosed with ischaemic or haemorrhagic stroke confirmed by brain imaging were included. Detailed information about EFFECTS can be found elsewhere. [24]

Study sample

The Väststroke dataset comprises data on 6,493 patients; the EFFECTS dataset comprises data on 1,500 patients (Supplementary Figure). The two datasets will be merged.

The inclusion criteria are as follows:

- age ≥18 years at stroke onset;

For peer review only - http://bmjopen.bmj.com/site/about/guidelines.xhtml

- a diagnosis of stroke according to the International Classification of Diseases (ICD-10): intracerebral haemorrhage (I61), cerebral infarction (I63), and stroke, not specified as haemorrhage or infarction (I64);
- patients with a previous stroke and/or transient ischemic attack (TIA);
- a complete MoCA.

The exclusion criterion was as follows:

- duplicate registration on the Väststroke and EFFECTS datasets (duplicates will be removed from the Väststroke dataset).

For peer review only

Study procedure

Väststroke data were registered by healthcare professionals working in stroke units. The MoCA was administered by occupational therapists. Occupational therapists at stroke units have regular workshops and peer reviews regarding the administration and interpretation of the MoCA. In the EFFECTS, the patients were enrolled in the stroke and rehabilitation units between 2 and 15 days after stroke onset. Cognitive screening was performed by study personnel such as nurses or physicians at the local centre, without any formal training of the instrument; sometimes, the assessment by the local occupational therapist was used. Detailed information about the EFFECTS study is available elsewhere. [26] Cognitive screening was performed during the acute and early subacute phases of stroke.

Study variables

Only the variables available in both the Väststroke and EFFECTS datasets will be used. Detailed information on all variables that will be included in the study, their categories, and coding for the merged datasets are presented in Supplementary Table 1.

The MoCA is a valid and reliable instrument for cognitive screening in the acute and subacute phases of stroke.[8-10] The total MoCA score ranges between 0 and 30 points (p); 1 p for a maximum of 12 years of education can be added to the total score. A score of ≤ 25 p indicates cognitive impairment.[8] MoCA scores were registered in the datasets as follows:

- orientation (range, 0−6 p);
- delayed recall (range, 0−5 p);
- visuospatial/executive functions (range, 0–5 p): trial (1 p) + cube (1 p) + clock (3 p);

For peer review only - http://bmjopen.bmj.com/site/about/guidelines.xhtml

- naming of three animals (range, 0 − 3 p);
- digit span, two tasks (range, 0−2 p);
- repetition of two sentences (range, 0–2 p);
- categories, two tasks (range, 0−2 p);
- serial 7 (range, 0−3 p);
- fluency (range, 0−1 p);
- tap on A (range, 0−1 p).

Stroke severity at onset was assessed using the National Institutes of Health Stroke Scale (NIHSS).[27] The NIHSS comprises 15 items with varying score ranges per item. The total NIHSS score is 0−42 p, with a higher score indicating a more severe stroke. [27]

Reperfusion treatment will be categorised as both thrombolysis and thrombectomy, only thrombolysis, or only thrombectomy.

Information about a previous stroke and/or TIA was recorded (yes, no, or unknown). The Väststroke register comprises patients with a first-ever stroke. In the EFFECTS study, patients could have a prior stroke and/or TIA, and these two conditions could not be distinguished.

Aggregated variables

The T-MoCA is a short version of the MoCA that is based on the following items: delayed recall, digit span, list letters, serial 7, sentence repetition, fluency, abstraction, and orientation.[14] The total T-MoCA score is 22 p. The reported threshold for impaired cognition is ≤18 p.[14]

The NINDS-CSN is based on items such as delayed recall, fluency, and orientation.[15, 17-19] The total NINDS-CSN score is 12 p. The suggested thresholds for impaired cognition are ≤9 p,[13, 15] ≤10 p,[16] and ≤6 p.[17, 28]

The SF-MoCA is based on items such as delayed recall, serial 7, and orientation.[11, 12] The total SF-MoCA score is 14 p, and a cutoff of ≤8 p has been suggested as an indicator for impaired cognition. [11, 12]

Statistics

Data will be presented as the mean and standard deviation, median and interquartile range, minimum–maximum (min–max), number (n), and proportion (%) (Supplementary Tables 2 and 3). The odds ratio and 95% confidence interval (CI) will be presented when appropriate. The

For peer review only - http://bmjopen.bmj.com/site/about/guidelines.xhtml

significance level for the statistical tests will be set at α = 5%. All statistical tests will be two-tailed.

Objective 1

Two different approaches will be used to identify the MoCA items that have the potential to form the s-MoCA-SWE: exploratory factor analysis[29, 30] and boosted regression tree.[31]

Exploratory factor analysis is a dimension-reduction method with good sensitivity for identifying the latent connections between variables. Exploratory factor analysis will be performed in several steps. First, the correlations between the MoCA items will be calculated. Second, the results of Bartlett's and Kaiser–Meyer–Olkin tests will be evaluated; values lower than 0.05 and greater than 0.7, respectively, are desirable. Third, the number of factors will be determined based on the scree plot and parallel analysis scree plot of eigenvalues. A factor-extraction method will be chosen, and a decision about rotation will be made based on the results of the previous steps. MoCA items with loading >0.6 will be chosen as having the potential to enter the s-MoCA-SWE.[30]

Boosted regression tree is a supervised machine-learning algorithm. In the boosted regression tree algorithm, modelling trees are grown sequentially (e.g. each tree is grown based on the information from the previously grown trees and each tree is fit to the residuals from the previous tree). For this analysis, the dataset will be split into training (80%) and test (20%) datasets. The s-MoCA-SWE boosted regression tree will be developed based on the training dataset, and tuning parameters such as the number of trees (B), shrinkage parameter (λ), and interaction depths (d) will be chosen based on 10-fold cross-validation and bootstrapping. The MoCA items with the potential to build an s-MoCA-SWE will be chosen based on the boosted regression tree model with adjusted tuning parameters and variable coefficients >5.0. The s-MoCA-SWE developed based on the training dataset will be further tested on the test dataset.

The decision between the set of MoCA items derived from exploratory factor analysis and that derived from the boosted regression tree algorithm will be made by applying binary logistic regression analysis. The sets will be entered as independent variables and tested using different regression models. The dependent variable will be dichotomised MoCA (≤25 p for impaired cognition).[8, 32] The s-MoCA-SWE version with the highest sensitivity and classification accuracy for cognitive impairment will be analysed in Objective 2.

For peer review only - http://bmjopen.bmj.com/site/about/guidelines.xhtml

Objective 2

To find the optimal cutoff value of s-MoCA-SWE for classifying patients with and without cognitive impairment, binary logistic regression analysis will be performed. The MoCA cutoff of ≤25 p for impaired cognition will be the reference output,[8, 32] and the s-MoCA-SWE full score will be the independent variable. The cutoff value of s-MoCA-SWE will be chosen based on its good sensitivity for identifying patients with cognitive impairment.

Objective 3

Four cross tables will be evaluated. The reference variable will be dichotomised MoCA (≤25 p for impaired cognition).[8, 32] The index variables will be dichotomised s-MoCA-SWE (according to the results from Objective 2), T-MoCA (≤18 p for impaired cognition),[14] NINDS-CSN (≤9 p for impaired cognition),[13, 15] and SF-MoCA (≤8 p for impaired cognition).[11, 12] The psychometric measurements of sensitivity, specificity, positive predictive value, negative predictive value, and Youden's index with 95% CI will be evaluated.

Receiving operating characteristic curve analysis will be performed. The full scores of s-MoCA-SWE, T-MoCA, NINDS-CSN, and SF-MoCA will be entered as test variables, and the dichotomised MoCA (≤25 p for impaired cognition) will be entered as the state variable.[8, 32] The areas under the curve will be interpreted as follows: 0.7–0.9, moderate accuracy and 0.5–0.7, low accuracy.[33]

Subgroup analyses

The properties of the s-MoCA-SWE will be evaluated further. Subgroup analysis will be performed by studying the differences between patients with and without a previous stroke/TIA, with the hypothesis that patients with a previous stroke are more likely to have impaired cognition; by stroke severity, with the hypothesis that patients with more severe stroke are more likely to have more impaired cognition; and by age, with the hypothesis that older patients are more likely to have impaired cognition.

Missing data

For descriptive statistics, data on all included patients will be used. The proportion of missing values will be provided for each baseline variable.

For peer review only - http://bmjopen.bmj.com/site/about/guidelines.xhtml

Statistical software

Data will be analysed using SPSS Statistics for Windows version 27.0 (released on 2018; IBM Corp., Armonk, NY) and R version 4.0.2 (R Core Team. R: A language and environment for statistical computing. R Foundation for Statistical Computing, Vienna, Austria.).

Patient and public involvement statement

The patients and the public were not involved in the design of the protocol.

Cognition has been identified by patients, their caregivers, and healthcare professionals as one of the most important research areas in stroke. [34] Furthermore, many patients have reported unmet needs regarding problems with cognitive functions, such as memory, concentration, and speaking/reading. [35] Thus, early identification of cognitive impairment is important.

Patients with stroke can find cognitive assessments fairly burdensome in acute stroke and would prefer short tests if possible. By developing s-MoCA-SWE, we hope to have a simpler and more efficient cognitive screening tool that can be used during acute and subacute stroke. Theoretically, this would lead to a higher proportion of patients who receive the recommended cognitive screenings.

ETHICS AND DISSEMINATION

Väststroke: The study obtained ethics approval from the Regional Ethical Review Board in Gothenburg (346–16) and the Swedish Ethical Review Authority (amendment 2019–04299). According to the Swedish Data Protection Authority, the handling of data generated within the framework of quality registers represents an exception to the general rule of written informed consent being required from patients. Furthermore, the Personal Data Act (Swedish law #1998:204, issued 29 April 1998) allows data from medical charts to be collected for clinical purposes and quality control without written informed consent.

The EFFECTS study has received ethical approval from the Stockholm Ethics Committee (2013/1265-31/2 on 30/09/2013). All participants of the EFFECTS study provided written consent for inclusion in the randomised controlled trial. They also provided written and oral consent to merge their data with other registry data.

For peer review only - http://bmjopen.bmj.com/site/about/guidelines.xhtml

In order to disseminate the study findings to a wide audience, results will be published in international, peer-reviewed journals, presented at local and international conferences, communicated to clinical practitioners in local meetings and seminars, and shared via social media channels.[36] Furthermore, a short report will be published in Swedish journals that target professionals who perform clinical cognitive screenings.

COMPETING INTERESTS

None declared.

FUNDING

This work was supported by the IRIS Foundation, Swedish Research Council (VR2017-00946), Swedish Heart and Lung Foundation, Swedish Brain Foundation, Promobilia, Swedish state under an agreement between the Swedish government and the county councils, ALF agreement (ALFGBG-718711, ALFGBG-877961), Swedish National Stroke Association, Local Research and Development Board for Gothenburg and Södra Bohuslän, Greta and Einar Asker's Foundation, Rune and Ulla Almöv's Foundation for Neurological Research, Hjalmar Svensson's Research Foundation, P-O Ahl & J-B Wennerström's Foundation; Herbert & Karin Jacobssons Foundation, and Sahlgrenska University Hospital Foundations.

AUTHOR CONTRIBUTIONS

TA: conceptualisation of the study, statistics, drafting of the manuscript; EL: chief investigator of EFFECTS, providing of EFFECTS's data, critical revision of the manuscript; DB: statistical consultations, critical revision of the manuscript; ME: statistical consultations, critical revision of the manuscript; TQ: statistical consultations, conceptualisation of the study, critical revision of the manuscript; KSS: acquisition of Väststroke data, conceptualisation of the study, critical revision of the manuscript.

DATA AVAILABILITY STATEMENT

According to the Swedish regulations shown at https://etikprovning.se/for-forskare/ansvar/, the data cannot be publicly shared because of ethical and legal reasons. Researchers can request access to the data by emailing the principal investigators Katharina S. Sunnerhagen at

For peer review only - http://bmjopen.bmj.com/site/about/guidelines.xhtml

ks.sunnerhagen@neuro.gu.se (Väststroke) and Erik Lundström at erik.lundstrom@neuro.uu.se (EFFECTS).

References

1. McDonald MW, Black SE, Copland DA, et al. Cognition in stroke rehabilitation and recovery research: Consensus-based core recommendations from the second Stroke Recovery and Rehabilitation Roundtable. *Int J Stroke* 2019;**14**:774–782.
2. Sachdev PS, Blacker D, Blazer DG, et al. Classifying neurocognitive disorders: The DSM-5 approach. *Nat Rev Neurol* 2014;**10**:634–642.
3. Nys GMS, Van Zandvoort MJE, De Kort PLM et al. Cognitive disorders in acute stroke: Prevalence and clinical determinants. *Cerebrovasc Dis* 2007;**23**:408–416.
4. Abzhandadze T, Reinholdsson M, Sunnerhagen KS. NIHSS is not enough for cognitive screening in acute stroke: A cross-sectional, retrospective study. *Sci Rep* 2020;**10**: doi: 10.1038/s41598-019-57316-8.
5. Patel MD, Coshall C, Rudd AG et al. Cognitive impairment after stroke: Clinical determinants and its associations with long-term stroke outcomes. *J Am Geriatr Soc* 2002;**50**:700–706.
6. Eng JJ, Bird ML, Godecke E, et al. Moving stroke rehabilitation research evidence into clinical practice: Consensus-based core recommendations from the Stroke Recovery and Rehabilitation Roundtable. *Int J Stroke* 2019;**14**:766–773.
7. Quinn TJ, Elliott E, Langhorne P. Cognitive and mood assessment tools for use in stroke. *Stroke* 2018;**49**:483–490.
8. Nasreddine ZS, Phillips NA, Bédirian V, et al. The Montreal Cognitive Assessment, MoCA: A brief screening tool for mild cognitive impairment. *J Am Geriatr Soc* 2005;**53**:695–699.
9. Munthe-Kaas R, Aam S, Saltvedt I, et al. Test accuracy of the Montreal cognitive assessment in screening for early poststroke neurocognitive disorder: The nor-COAST study. *Stroke* 2021;**52**:317–320.
10. Chiti G, Pantoni L. Use of Montreal cognitive assessment in patients with stroke. *Stroke* 2014;**45**:3135–3140.
11. Horton DK, Hynan LS, Lacritz LH et al. An abbreviated Montreal cognitive assessment (MoCA) for dementia screening. *Clin Neuropsychol* 2015;**29**:413–425.
12. McDicken JA, Elliott E, Blayney G, et al. Accuracy of the short-form Montreal Cognitive Assessment: Systematic review and validation. *Int J Geriatr Psychiatry* 2019;**34**:1515–1525.
13. Pendlebury ST, Welch SJ, Cuthbertson FC et al. Telephone assessment of cognition after transient ischemic attack and stroke: Modified telephone interview of cognitive status and telephone Montreal Cognitive Assessment versus face-to-face Montreal Cognitive Assessment and neuropsychological battery. *Stroke* 2013;**44**:227–229.
14. Wittich W, Phillips N, Nasreddine ZS et al. Sensitivity and specificity of the Montreal cognitive assessment modified for individuals who are visually impaired. *J Vis Impairment Blindness* 2010;**104**:360–368.
15. Cameron JD, Gallagher R, Pressler SJ, et al. Sensitivity and specificity of a five-minute cognitive screening test in patients with heart failure. *J Card Fail* 2016;**22**:99–107.
16. Kaur D, Kumar G, Singh AK. Quick screening of cognitive function in Indian multiple sclerosis patients using Montreal cognitive assessment test-short version. *Ann Indian Acad Neurol* 2013;**16**:585–589.

For peer review only - http://bmjopen.bmj.com/site/about/guidelines.xhtml

17. Lim JS, Oh MS, Lee JH, et al. Prediction of post-stroke dementia using NINDS-CSN 5-minute neuropsychology protocol in acute stroke. *Int Psychogeriatr* 2017;**29**:777–784.
18. Lin HF, Chern CM, Chen HM, et al. Validation of NINDS-VCI neuropsychology protocols for vascular cognitive impairment in Taiwan. *PLoS One*. Public Library of Science 2016;11: doi:10.1371/journal.pone.0156404
19. Wei J, Jin X, Chen B, et al. Comparative study of two short-form versions of the Montreal cognitive assessment for screening of post-stroke cognitive impairment in a Chinese population. *Clin Interv Aging* 2020;**15**:907–914.
20. Wong A, Law LS, Liu W et al. Montreal cognitive assessment: One cutoff never fits all. *Stroke* 2015;**46**:3547–3550.
21. Stern Y. What is cognitive reserve? Theory and research application of the reserve concept. *J Int Neuropsychol Soc* 2002;**8**:448–460.
22. Shin M, Sohn MK, Lee J et al. Effect of cognitive reserve on risk of cognitive impairment and recovery after stroke: The KOSCO Study. *Stroke* 2020;**51**:99–107.
23. O'Driscoll C, Shaikh M. Cross-cultural applicability of the Montreal cognitive assessment (MoCA): A systematic review. *J Alzheimers Dis* 2017;**58**:789–801.
24. EFFECTS Trial Collaboration. Safety and efficacy of fluoxetine on functional recovery after acute stroke (EFFECTS): A randomised, double-blind, placebo-controlled trial. *Lancet Neurol* 2020;**19**:661–669.
25. Asplund K, Eriksson M, Riks-Stroke Collaboration. Inflammation, poststroke depression and statins. *Int J Stroke* 2011;**6**:567–568.
26. Bernhardt J, Hayward KS, Kwakkel G, et al. Agreed definitions and a shared vision for new standards in stroke recovery research: The Stroke Recovery and Rehabilitation Roundtable taskforce. *Int J Stroke* 2017;**12**:444–450.
27. Kasner SE, Chalela JA, Luciano JM, et al. Reliability and validity of estimating the NIH stroke scale score from medical records. *Stroke* 1999;**30**:1534–1537.
28. Dong Y, Xu J, Chan BP-L, et al. The Montreal Cognitive Assessment is superior to National Institute of Neurological Disease and Stroke-Canadian Stroke Network 5-minute protocol in predicting vascular cognitive impairment at 1 year. *BMC Neurol* 2016;**16**: doi: 10.1186/s12883-016-0570-y.
29. Child D. Essentials of factor analysis. 3. *Educ Educ*. New York: NY: Continuum 2006.
30. Kline P. An easy guide to factor analysis. *London: london*. Routledge 1994.
31. Chen T, Guestrin C. *XGBoost: A scalable tree boosting system. Ithaca2016*.
32. Borland E, Nägga K, Nilsson PM et al. The Montreal cognitive assessment: Normative data from a Large Swedish population-based cohort. *J Alzheimers Dis* 2017;**59**:893–901.
33. Fischer JE, Bachmann LM, Jaeschke R. A readers' guide to the interpretation of diagnostic test properties: Clinical example of sepsis. *Intensive Care Med* 2003;**29**:1043–1051.
34. Pollock A, St George B, Fenton M et al. Top 10 research priorities relating to life after stroke - Consensus from stroke survivors, caregivers, and health professionals. *Int J Stroke* 2014;**9**:313–320.
35. McKevitt C, Fudge N, Redfern J, et al. Self-reported long-term needs after stroke. *Stroke* 2011;**42**:1398–1403.
36. Edwards DJ. Dissemination of research results: On the path to practice change. *Can J Hosp Pharm* 2015;**68**:465–469.

For peer review only - http://bmjopen.bmj.com/site/about/guidelines.xhtml

Supplementary File

Development of a short-form Swedish version of the Montreal Cognitive Assessment (s-MoCA-SWE): Protocol for a cross-sectional study

Tamar Abzhandadze[1,2], Erik Lundström[3], Dongni Buvarp[1], Marie Eriksson[4], Terence J. Quinn [5], Katharina S. Sunnerhagen[1,6].

[1]Institute of Neuroscience and Physiology, The Sahlgrenska Academy, University of Gothenburg, Gothenburg, Sweden.

[2]Department of Occupational Therapy and Physiotherapy, Sahlgrenska University Hospital, Gothenburg, Sweden.

[3]Department of Neuroscience, Neurology, Uppsala University, Akademiska Sjukhuset, Uppsala, Sweden.

[4]Department of Statistics, USBE, Umeå University, Umeå, Sweden.

[5]Institute of Cardiovascular and Medical Sciences, University of Glasgow, Glasgow, UK.

[6] Neurocare, Sahlgrenska University Hospital, Gothenburg, Sweden.

Correspondence to:

Tamar Abzhandadze

Institute of Neuroscience and Physiology

Rehabilitation Medicine, University of Gothenburg

Per Dubbsgatan 14, fl. 3

413 45 Gothenburg, Sweden

E-mail: tamar.abzhandadze@gu.se, tamar.abzhandadze@vgregion.se

For peer review only

Supplementary Table 1. Description of variables and their categories respective data set and in the merged dataset.

**Väststroke**	**Väststroke: variable categories and coding**	**EFFECTS dataset**	**EFFECTS: variable categories and coding**	**Merged dataset: variable names and coding**
				Cohorts, binary:<br>1 = Väststroke<br>2 = EFFECTS
Sex	Binary:<br>0 = female<br>1 = male	Pat_Gender	F = female<br>M = male	Sex, binary:<br>1 = female<br>2 = male
Age	Scale: range	AGE	Numeric	Age: numeric
Living arrangement/living alone prior to stroke	Binary:<br>1 = no<br>2 = yes	pat_Living_Arrangements	A = living alone<br>B = institutional living<br>C = other living arrangement<br>D = living with someone else	Living arrangement prior to stroke, binary:<br>1 = lived alone (Väststroke = 2, EFFECTS = A)<br>2 = did not live alone (Väststroke = 1, EFFECTS ≥B)
Needing help/dependent prior to stroke	Binary:<br>1 = no<br>2 = yes	Stroke daily	N = no<br>Y = yes<br><br>*Note: "N" means that the patient did not require assistance with ADLs prior to stroke.*	Needs assistance with ADL before stroke, binary:<br>1 = no<br>2 = yes
Diabetes	Binary:<br>0 = no<br>1 = yes	DIABETES	N = no<br>Y = yes<br>U = unknown	Diabetes, nominal:<br>0 = no<br>1 = yes<br>2 = unknown

For peer review only

TIA prior to stroke	Binary:<br>0 = no<br>1 = yes	PREV_Stroke_TIA<br><br>*Note: In EFFECTS, previous TIA and previous ischemic stroke are not distinguished.*	N = no<br>Y = yes<br>U = unknown	Previous TIA or ischemic stroke, nominal:<br>0 = no<br>1 = yes<br>2 = unknown
Stroke diagnosis	Nominal, 3 levels:<br>1 = I61<br>2 = I63<br>3 = I64	Diagnosis	1 = ischemic stroke<br>2 = intracerebral haemorrhage<br>3 = non-stroke	Stroke diagnosis, nominal:<br>1 = I63<br>2 = I61<br>3 = I64
Reperfusion	1 = both IVT and endovascular thrombectomy<br>2 = endovascular thrombectomy only<br>3 = IVT only	Thrombolysis_Trombectomy	1 = both IVT and endovascular thrombectomy<br>2 = endovascular thrombectomy only<br>3 = IVT only	Reperfusion treatment, nominal:<br>1 = both IVT and endovascular thrombectomy<br>2 = endovascular thrombectomy only<br>3 = IVT only
NIHSS awareness	Ordinal: range 0–3	Stroke_NIHSS_1a	0 = alert<br>1 = not alert, but arousable with minimal stimulation<br>2 = not alert, requires repeated stimulation<br>3 = coma	NIHSS 1a, awareness, ordinal:<br>0 = alert<br>1 = not alert, but arousable with minimal stimulation<br>2 = not alert, requires repeated stimulation<br>3 = coma
NIHSS orientation	Ordinal: range 0–2	Stroke_NIHSS_1b	0 = answers both correctly<br>1 = answers one correctly<br>2 = answers both incorrectly	NIHSS 1b, orientation, ordinal:<br>0 = answers both correctly<br>1 = answers one correctly<br>2 = answers both incorrectly

For peer review only

NIHSS understanding	Ordinal: range 0–2	Stroke_NIHSS_1c	0 = obeys both correctly<br>1 = obeys one correctly<br>2 = obeys both incorrectly	NIHSS 1c, understanding, ordinal:<br>0 = obeys both correctly<br>1 = obeys one correctly<br>2 = obeys both incorrectly
NIHSS eye movement	Ordinal: range 0–2	Stroke_NIHSS_2	0 = normal<br>1 = partial gaze palsy<br>2 = total gaze paresis	NIHSS 2, eye movement, ordinal:<br>0 = normal<br>1 = partial gaze palsy<br>2 = total gaze paresis
NIHSS visual field	Ordinal: range 0–3	Stroke_NIHSS_3	0 = no visual field loss<br>1 = partial hemianopia<br>2 = complete hemianopia<br>3 =bilateral hemianopia	NIHSS 3, visual field, ordinal:<br>0 = no visual field loss<br>1 = partial hemianopia<br>2 = complete hemianopia<br>3 = bilateral hemianopia
NIHSS facial palsy	Ordinal: range 0–3	Stroke_NIHSS_4	0 = normal symmetrical movement<br>1 = minor paralysis<br>2 = partial paralysis<br>3 = complete paralysis	NIHSS 4, facial palsy, ordinal:<br>0 = normal symmetrical movement<br>1 = minor paralysis<br>2 = partial paralysis<br>3 = complete paralysis
NIHSS motor arm: right	Ordinal: range 0–4	Stroke_NIHSS_7	0 = normal<br>1 = drift<br>2 = some effort against gravity<br>3 = no effort against gravity<br>4 = no effort against gravity	NIHSS 5, right motor arm, ordinal:<br>0 = normal<br>1 = drift<br>2 = some effort against gravity<br>3 = no effort against gravity<br>4 = no effort against gravity

For peer review only

NIHSS motor arm: left	Ordinal: range 0–4	Stroke_NIHSS_8	0 = normal<br>1 = drift<br>2 = some effort against gravity<br>3 = no effort against gravity<br>4 = no effort against gravity	NIHSS 6, left motor arm, ordinal:<br>0 = normal<br>1 = drift<br>2 = some effort against gravity<br>3 = no effort against gravity<br>4 = no effort against gravity
NIHSS motor leg: right	Ordinal: range 0–4	Stroke_NIHSS_5	0 = normal<br>1 = drift<br>2 = some effort against gravity<br>3 = no effort against gravity<br>4 = no effort against gravity	NIHSS 7, right motor leg, ordinal:<br>0 = normal<br>1 = drift<br>2 = some effort against gravity<br>3 = no effort against gravity<br>4 = no effort against gravity
NIHSS motor leg: left	Ordinal: range 0–4	Stroke_NIHSS_6	0 = normal<br>1 = drift<br>2 = some effort against gravity<br>3 = no effort against gravity<br>4 = no effort against gravity	NIHSS 8, left motor leg, ordinal:<br>0 = normal<br>1 = drift<br>2 = some effort against gravity<br>3 = no effort against gravity<br>4 = no effort against gravity
NIHSS ataxia	Ordinal: range 0–2	Stroke_NIHSS_9	0 = no ataxia<br>1 = present in one limb<br>2 = present in two limbs	NIHSS 9, ataxia, ordinal:<br>0 = no ataxia<br>1 = present in one limb<br>2 = present in two limbs
NIHSS sensory	Ordinal: range 0–2	Stroke_NIHSS_10	0 = normal<br>1 = mild-to-moderate sensory loss<br>2 = severe or total sensory loss	NIHSS 10, sensory, ordinal:<br>0 = normal<br>1 = mild-to-moderate sensory loss<br>2 = severe or total sensory loss

For peer review only

NIHSS language	Ordinal: range 0–3	Stroke_NIHSS_11	0 = no aphasia<br>1 = mild-to-moderate aphasia<br>2 = severe aphasia<br>3 = mute	NIHSS 11, language, ordinal:<br>0 = no aphasia<br>1 = mild-to-moderate aphasia<br>2 = severe aphasia<br>3 = mute
NIHSS dysarthria	Ordinal: range 0–2	Stroke_NIHSS_12	0 = normal<br>1 = mild-to-moderate dysarthria<br>2 = severe dysarthria<br>3 = untestable	NIHSS 12, dysarthria, ordinal:<br>0 = normal<br>1 = mild-to-moderate dysarthria<br>2 = severe dysarthria<br>3 = untestable
NIHSS neglect	Ordinal: range 0–2	Stroke_NIHSS_13	0 = normal<br>1 = inattention to one modality<br>2 = severe hemi-inattention	NIHSS 13, neglect, ordinal:<br>0 = normal<br>1 = inattention to one modality<br>2 = severe hemi-inattention
NIHSS total score	Ordinal: range 0–36	NIHSS	Ordinal: range 0–26	NIHSS total score, ordinal: range 0–36
MoCA visuospatial/executive functions: 1A + cube + clock	Ordinal: range 0–5	MOCA_Base_Viso_exe	Ordinal: range 0–5	MoCA visuospatial/executive functions: trial + cube + clock, ordinal: range 0–5
MoCA task: naming	Ordinal: range 0–3	MOCA_Base_Name	Ordinal: range 0–3	MoCA: naming, ordinal: range 0–3
MoCA attention task: numbers forward and backward	Ordinal: range 0–2	MOCA_Base_Att_fwd_back	Ordinal: range 0–2	MoCA: digit span, ordinal: range 0–2
MoCA attention task: knock on A	Nominal:<br>0 = no point<br>1 = 1 point	MOCA_Base_Att_knock	Nominal:<br>0 = no point<br>1 = 1 point	MoCA: tap on A, binary:<br>0 = no point<br>1 = 1 point
MoCA attention task: subtraction 100 - 7	Ordinal: range 0–3	MOCA_Base_Att_subtract	Ordinal: range 0–3	MoCA: serial 7, ordinal: range 0–3

For peer review only

MoCA language task: sentence repetition, 2	Ordinal: range 0–2	MOCA_Base_Lang_rep	Ordinal: range 0–2	MoCA: repetition, ordinal: range 0–2
MoCA language task: fluency, F word	Nominal:<br>0 = no point<br>1 = 1 point	MOCA_Base_Fluency	Nominal:<br>0 = no point<br>1 = 1 point	MoCA: fluency, binary:<br>0 = no point<br>1 = 1 point
MoCA abstraction task: categorizing of two tasks	Ordinal: range 0–2	MOCA_Base_Abstraction	Ordinal: range 0–2	MoCA: abstraction, ordinal: range 0–2
MoCA memory task: recall of five words	Ordinal: range 0–5	MOCA_Base_Delay	Ordinal: range 0–5	MoCA: delayed recall, ordinal: range 0–5
MoCA orientation task: six questions	Ordinal: range 0–6	MOCA_Base_Orientation	Ordinal: range 0–6	MoCA: orientation, ordinal: range 0–6
MoCA total score	Ordinal: range 1–30	MOCA_Base_Total	Ordinal: range 1–30	MoCA total score, ordinal: range 1–30
MoCA school ≤12 years, +1 point	Binary:<br>0 = no<br>1 = yes, +1 point	MOCA_Education	0 = no<br>1 = yes, +1 point	MoCA education, binary:<br>0 = no,<br>1 = yes, +1 point
				A telephone version of the MoCA, ordinal: range 0–22<br><br>Aggregated score of the MoCA items: delayed recall, digit span, list letters, serial 7, sentence repetition, fluency, abstraction, and orientation
				Short-form MoCA, ordinal: range 0–14<br><br>Aggregated score of the MoCA items: delayed recall, serial 7, and orientation

For peer review only

				NINDS-CSN–MoCA, ordinal: range 0–12<br><br>Aggregated score of the MoCA items: delayed recall, fluency, and orientation
Discharge destination*	Ordinal, 10 levels:<br>1 = own accommodation<br>2 = arranged accommodation<br>4 = another acute-care department<br>5 = geriatrics/rehabilitation<br>6 = deceased during treatment<br>7 = other acute-care department (e.g. the patient is resident in another country)<br>9 = not known<br>11 = still in hospital<br>12 = another stroke unit for aftercare<br>13 = health centre with emergency beds	Discharge residence	1 = own home<br>2 = relatives' home<br>3 = residential home<br>4 = long-term NHS home<br>6 = another hospital<br>7 = other	Discharge destination, ordinal:<br>1 = own or relatives' home (Väststroke 1, EFFECTS 1 and 2)<br>2 = residential home (Väststroke 2, EFFECTS 3 and 4)<br>3 = other hospital (Väststroke 4, 5, 11, and 12, EFFECTS 6)<br>4 = other (Väststroke 7, 9, and 13, EFFECTS 7)

Abbreviations: ADL, activity of daily living; IVT, intravenous thrombolysis; MoCA, Montreal Cognitive Assessment; NIHSS, National Institutes of Health Stroke Scale; NINDS-CSN, National Institute of Neurological Disorders and Stroke–Canadian Stroke Network; TIA, transient ischemic attack.

For peer review only

Supplementary Table 2. Patient baseline characteristics (template table)

	Categories	Total sample N =	Väststroke N =	EFFECTS N =
Sex, n (%)	Male			
	Female			
Age, y, mean (SD)				
Living arrangement, n (%)	Lives alone			
	Living with someone else			
Education, 1 point for a maximum of 12 years, n (%)	Yes			
	No			
Assistance with ADLs prior to stroke, n (%)	No			
	Yes			
Having diabetes, n (%)	No			
	Yes			
	Unknown			
Previous TIA or ischemic stroke, n (%)	No			
	Yes			
	Unknown			
Stroke diagnosis, n (%)	I61			
	I63			
	I64			

For peer review only

Reperfusion treatment, n (%)	Both IVT and ET<br>ET only<br>IVT only			
NIHSS, median (min–max)				
MoCA, median (min–max)				
Discharge destination, n (%)	Own or relatives' home<br>Residential or long-term<br>NHS home<br>Other hospitals<br>Other			

Note: The sum may vary due to missing data.

Abbreviations: ADL, activity of daily living; IVT, intravenous thrombolysis; IQR, interquartile range; MoCA, Montreal Cognitive Assessment; NIHSS, National Institutes of Health Stroke Scale; NHS, National Health System; SD, standard deviation; ET, endovascular thrombectomy; TIA, transient ischemic attack.

Variables with missing values, n (%):

For peer review only

Supplementary Table 3. Description of cognitive functions (template table).

		Total sample<br>N =	Väststroke<br>N =	EFFECTS<br>N =
	Points	n (%)	n (%)	n (%)
Visuospatial/executive functions	0<br>1<br>2<br>3<br>4<br>5			
Naming three animals	0<br>1<br>2<br>3			
Digit span	0<br>1<br>2			
Tap on A	0<br>1			
Serial 7	0<br>1			

For peer review only

	2<br>3			
Repetition	0<br>1<br>2			
Fluency	0<br>1			
Abstraction	0<br>1<br>2			
Delayed recall	0<br>1<br>2<br>3<br>4<br>5			
Orientation	0<br>1<br>2<br>3<br>4			

For peer review only

	5<br>6			
MoCA score	Median (IQR)			
T-MoCA	Median (IQR)			
NINDS-CSN–MoCA	Median (IQR)			
SF-MoCA	Median (IQR)			

Abbreviations: IQR, interquartile range; MoCA, Montreal Cognitive Assessment (score range: 1–30); NINDS-CSN–MoCA, National Institute of Neurological Disorders and Stroke–Canadian Stroke Network MoCA (score range: 0–12); SF-MoCA, short-form MoCA (score range: 0–14); T-MoCA, a telephone version of the MoCA (score range: 0–22).

For peer review only

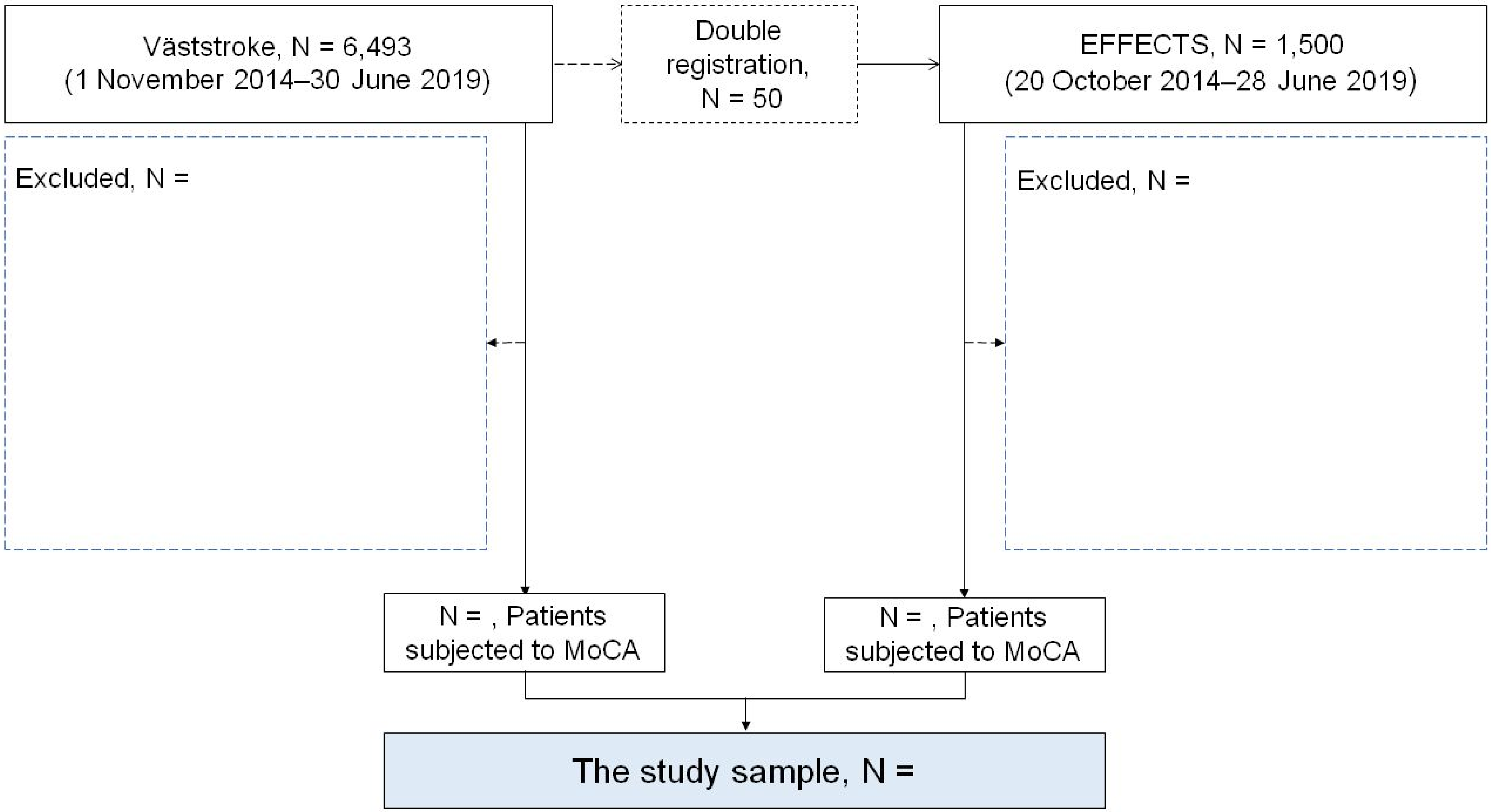

Supplementary Figure. Flowchart of patient selection (template diagram). Abbreviations: MoCA, the Montreal Cognitive Assessment; EFFECTS, **E**fficacy o**F** **F**luoxetine—a randomis**E**d **C**ontrolled **T**rial in **S**troke.

Development of a short-form Swedish version of the Montreal Cognitive Assessment (s-MoCA-SWE): Protocol for a cross-sectional study

Journal:	*BMJ Open*
Manuscript ID	bmjopen-2021-049035.R1
Article Type:	Protocol
Date Submitted by the Author:	03-Mar-2021
Complete List of Authors:	Abzhandadze, Tamar; University of Gothenburg Sahlgrenska Academy, Institute of Neuroscience and Physiology; Sahlgrenska University Hospital, Department of Occupational Therapy and Physiotherapy<br>Lundström, Erik; Akademiska Sjukhuset, Department of Neuroscience<br>Buvarp, Dongni; University of Gothenburg, Institute of Neuroscience and Physiology<br>Eriksson, Marie; Umeå University, Department of Statistics, USBE<br>Quinn, Terry; University of Glasgow, Cardiovascular and Medical Sciences<br>Sunnerhagen, Katharina; University of Gothenburg, Institute of Neuroscience and Physiology; Sahlgrenska University Hospital, Neurocare
**Primary Subject Heading**:	Neurology
Secondary Subject Heading:	Rehabilitation medicine, Research methods
Keywords:	STROKE MEDICINE, Stroke < NEUROLOGY, REHABILITATION MEDICINE, Delirium & cognitive disorders < PSYCHIATRY

SCHOLARONE™
Manuscripts

I, the Submitting Author has the right to grant and does grant on behalf of all authors of the Work (as defined in the below author licence), an exclusive licence and/or a non-exclusive licence for contributions from authors who are: i) UK Crown employees; ii) where BMJ has agreed a CC-BY licence shall apply, and/or iii) in accordance with the terms applicable for US Federal Government officers or employees acting as part of their official duties; on a worldwide, perpetual, irrevocable, royalty-free basis to BMJ Publishing Group Ltd ("BMJ") its licensees and where the relevant Journal is co-owned by BMJ to the co-owners of the Journal, to publish the Work in this journal and any other BMJ products and to exploit all rights, as set out in our licence.

The Submitting Author accepts and understands that any supply made under these terms is made by BMJ to the Submitting Author unless you are acting as an employee on behalf of your employer or a postgraduate student of an affiliated institution which is paying any applicable article publishing charge ("APC") for Open Access articles. Where the Submitting Author wishes to make the Work available on an Open Access basis (and intends to pay the relevant APC), the terms of reuse of such Open Access shall be governed by a Creative Commons licence – details of these licences and which Creative Commons licence will apply to this Work are set out in our licence referred to above.

Other than as permitted in any relevant BMJ Author's Self Archiving Policies, I confirm this Work has not been accepted for publication elsewhere, is not being considered for publication elsewhere and does not duplicate material already published. I confirm all authors consent to publication of this Work and authorise the granting of this licence.

For peer review only

Development of a short-form Swedish version of the Montreal Cognitive Assessment (s-MoCA-SWE): Protocol for a cross-sectional study

Tamar Abzhandadze[1,2], Erik Lundström[3], Dongni Buvarp[1], Marie Eriksson[4], Terence J. Quinn [5], Katharina S. Sunnerhagen[1,6].

[1]Institute of Neuroscience and Physiology, The Sahlgrenska Academy, University of Gothenburg, Gothenburg, Sweden.

[2]Department of Occupational Therapy and Physiotherapy, Sahlgrenska University Hospital, Gothenburg, Sweden.

[3]Department of Neuroscience, Neurology, Uppsala University, Akademiska Sjukhuset, Uppsala, Sweden.

[4]Department of Statistics, USBE, Umeå University, Umeå, Sweden.

[5]Institute of Cardiovascular and Medical Sciences, University of Glasgow, Glasgow, UK.

[6] Neurocare, Sahlgrenska University Hospital, Gothenburg, Sweden.

Correspondence to:

Tamar Abzhandadze

Institute of Neuroscience and Physiology

Rehabilitation Medicine, University of Gothenburg

Per Dubbsgatan 14, fl. 3

413 45 Gothenburg, Sweden

E-mail: tamar.abzhandadze@gu.se, tamar.abzhandadze@vgregion.se

Word count, excluding title page, abstract, references, figures, and tables: 2,647

For peer review only - http://bmjopen.bmj.com/site/about/guidelines.xhtml

ABSTRACT

Introduction

Short forms of the Montreal Cognitive Assessment (MoCA) have allowed quick cognitive screening. However, none of the available short forms has been created or validated in a Swedish sample of patients with stroke.

The aim is to develop a short-form Swedish version of the Montreal Cognitive Assessment (s-MoCA-SWE) in a sample of patients with acute and subacute stroke. The specific objectives are: (1) to identify a subgroup of MoCA items that have the potential to form the s-MoCA-SWE; (2) to determine the optimal cutoff value of s-MoCA-SWE for predicting cognitive impairment; (3) and to compare the psychometric properties of s-MoCA-SWE with those of previously developed MoCA short forms.

Methods and analysis

This is a statistical analysis protocol for a cross-sectional study. The study sample will comprise patients from Väststroke, a local stroke registry from Gothenburg, Sweden and **E**fficacy o**F** **F**luoxetine—a randomis**E**d **C**ontrolled **T**rial in **S**troke (EFFECTS), a randomised controlled trial in Sweden. The s-MoCA-SWE will be developed by using exploratory factor analysis and the boosted regression tree algorithm. The cutoff value of s-MoCA-SWE for impaired cognition will be determined based on binary logistic regression analysis. The psychometric properties of s-MoCA-SWE will be compared with those of other MoCA short forms by using cross-tabulation and area under the receiving operating characteristic curve analyses.

Ethics and dissemination

The Väststroke study has received ethical approval from the Regional Ethical Review Board in Gothenburg (346–16) and the Swedish Ethical Review Authority (amendment 2019–04299). The handling of data generated within the framework of quality registers does not require written informed consent from patients. The EFFECTS study has received ethical approval from the Stockholm Ethics Committee (2013/1265-31/2 on 30/09/2013). All participants provided written consent. Results will be published in an international, peer-reviewed journal, presented at conferences, and communicated to clinical practitioners in local meetings and seminars.

For peer review only - http://bmjopen.bmj.com/site/about/guidelines.xhtml

Key words: cognitive screening, acute and subacute stroke, statistical analyses plan, development, validation.

For peer review only

STRENGTHS AND LIMITATIONS OF THIS STUDY

- A short form of the Swedish version of the Montreal Cognitive Assessment will be created for the first time for use in patients with stroke.
- The study will use a large sample size from different acute stroke and stroke rehabilitation units across Sweden.
- Data will be analysed using various statistical approaches.
- The retrospective analysis of registry data is one limitation of the study.
- A high number of non-random missing Montreal Cognitive Assessment values is expected.

For peer review only

For peer review only - http://bmjopen.bmj.com/site/about/guidelines.xhtml

INTRODUCTION

Cognitive impairment, a common sequela of stroke, has been defined as a new cognitive deficit that develops in the first 3 months after stroke and cannot be explained by other conditions or diseases.[1] Cognitive impairment is a consequence of complex interactions between age, education, and injury size and location.[2] The prevalence of cognitive impairment in the first 3 weeks after stroke can vary between 55% and 59% depending on the time of assessment and the assessment instruments.[3, 4] Previous studies have shown that cognitive impairment after stroke is associated with higher mortality and long-term disability.[5] Thus, early identification of cognitive impairments after stroke is important for planning individually tailored rehabilitation.

Comprehensive cognitive assessments in acute stroke units can be time- and resource-demanding for patients as well as for healthcare professionals. Therefore, the use of short screening tools for cognitive impairment is recommended.[6, 7] The Montreal Cognitive Assessment (MoCA) is frequently used to assess cognitive function.[7] The MoCA targets cognitive domains such as visuospatial ability, executive function, attention, concentration, working memory, language, short-term memory, and orientation.[8] It has a good sensitivity for detecting post-stroke cognitive impairment.[9, 10] To make cognitive screenings time effective, several short forms of the MoCA have been developed and tested in recent years, including a telephone version of the MoCA (T-MoCA), a short version of the MoCA that has been designed by the National Institute of Neurological Disorders and Stroke and the Canadian Stroke Network (NINDS-CSN, 5-minute protocol), and the short-form MoCA (SF-MoCA).[11-19] Different cutoffs for impaired cognition have been reported for the MoCA as well as its short forms.[11, 12, 15, 17-20] None of the available short forms have been developed or validated in a Swedish sample of patients with stroke.

Performance on cognitive tests after stroke can depend on premorbid cognitive reserves, which are associated with education, occupation, and socioeconomic situation.[21, 22] Items of the MoCA can function differently in various cultures; therefore, cultural validation of the MoCA has been recommended for generalisability.[23] Theoretically, this recommendation is accurate even when it comes to short forms of the MoCA. In order to have a short form of the MoCA used in a broader context as a time-effective screening instrument with good sensitivity for detecting cognitive impairment, it needs to be validated culturally within the target population.

For peer review only - http://bmjopen.bmj.com/site/about/guidelines.xhtml

Study aims

The aim of this study is to develop a short-form Swedish version of the Montreal Cognitive Assessment (s-MoCA-SWE) using data from Swedish stroke cohorts. The specific objectives are: (1) to identify a subgroup of MoCA items that have the potential to form the s-MoCA-SWE; (2) to determine the optimal cutoff value of s-MoCA-SWE for predicting cognitive impairment; and (3) to compare the psychometric properties of s-MoCA-SWE with those of T-MoCA, NINDS-CSN, and SF-MoCA.

METHODS AND ANALYSIS

Study design

This is a cross-sectional, exploratory study. Two datasets will be used: Väststroke, a local stroke registry from Gothenburg, Sweden and **E**fficacy o**F** **F**luoxetine—a randomis**E**d **C**ontrolled **T**rial in **S**troke (EFFECTS), a randomised controlled trial.[24]

The Väststroke register comprises data from three stroke units in Sahlgrenska University Hospital. The Väststroke registry was linked to the Riksstroke, a national quality register for stroke care in Sweden, via each patient's unique personal identification number.[25] The statisticians at Riksstroke merged the data. The Väststroke data from 1 November 2014 to 30 June 2019 were retrieved.

The EFFECTS was a randomized, double-blind, placebo-controlled clinical trial conducted in 35 stroke or rehabilitation units in Sweden.[24] The inclusion period was from 20 October 2014 to 28 June 2019.[24] Briefly, patients aged ≥ 18 years and diagnosed with ischaemic or haemorrhagic stroke confirmed by brain imaging were included. Detailed information about EFFECTS can be found elsewhere. [24]

Study sample

The Väststroke dataset comprises data on 6,493 patients; the EFFECTS dataset comprises data on 1,500 patients (Supplementary Figure). The two datasets will be aligned.

The inclusion criteria are as follows:

- age ≥18 years at stroke onset;

For peer review only - http://bmjopen.bmj.com/site/about/guidelines.xhtml

- a diagnosis of stroke according to the International Classification of Diseases (ICD-10): intracerebral haemorrhage (I61), cerebral infarction (I63), and stroke, not specified as haemorrhage or infarction (I64);
- patients with a previous stroke and/or transient ischemic attack (TIA);
- a complete MoCA (0–30 p).

The exclusion criterion was as follows:

- duplicate registration on the Väststroke and EFFECTS datasets (duplicates will be removed from the Väststroke dataset).

Study procedure

Väststroke data were registered by healthcare professionals working in stroke units. The MoCA was administered by occupational therapists. Occupational therapists at stroke units have regular workshops and peer reviews regarding the administration and interpretation of the MoCA. In the EFFECTS, the patients were enrolled in the stroke and rehabilitation units between 2 and 15 days after stroke onset. Cognitive screening was performed by study personnel such as nurses or physicians at the local centre, without any formal training of the instrument; sometimes, the assessment by the local occupational therapist was used. Detailed information about the EFFECTS study is available elsewhere. [26] Cognitive screening was performed during the acute and early subacute phases of stroke.

Study variables

Only the variables available in both the Väststroke and EFFECTS datasets will be used. Detailed information on all variables that will be included in the study, their categories, and coding for the merged datasets are presented in Supplementary Table 1.

The MoCA is a valid and reliable instrument for cognitive screening in the acute and subacute phases of stroke.[8-10] The total MoCA score ranges between 0 and 30 points (p); 1 p for a maximum of 12 years of education can be added to the total score. A score of ≤ 25 p indicates cognitive impairment.[8] MoCA scores were registered in the datasets as follows:

- orientation (range, 0−6 p);
- delayed recall (range, 0−5 p);
- visuospatial/executive functions (range, 0–5 p): trial (1 p) + cube (1 p) + clock (3 p);

For peer review only - http://bmjopen.bmj.com/site/about/guidelines.xhtml

- naming of three animals (range, 0 − 3 p);
- digit span, two tasks (range, 0−2 p);
- repetition of two sentences (range, 0–2 p);
- categories, two tasks (range, 0−2 p);
- serial 7 (range, 0−3 p);
- fluency (range, 0−1 p);
- tap on A (range, 0−1 p).

Stroke severity at onset was assessed using the National Institutes of Health Stroke Scale (NIHSS). [27] The NIHSS comprises 15 items with varying score ranges per item. The total NIHSS score is 0−42 p, with a higher score indicating a more severe stroke. [27]

Reperfusion treatment will be categorised as both thrombolysis and thrombectomy, only thrombolysis, or only thrombectomy.

Information about a previous stroke and/or TIA was recorded (yes, no, or unknown). The Väststroke register comprises patients with a first-ever stroke, however patients with previous TIA were included. In the EFFECTS study, patients could have a prior stroke and/or TIA, and these two conditions could not be distinguished.

Aggregated variables

The T-MoCA is a short version of the MoCA that is based on the following items: delayed recall, digit span, list letters, serial 7, sentence repetition, fluency, abstraction, and orientation.[14] The total T-MoCA score is 22 p. The reported threshold for impaired cognition is ≤18 p.[14]

The NINDS-CSN is based on items such as delayed recall, fluency, and orientation.[15, 17-19] The total NINDS-CSN score is 12 p. The suggested thresholds for impaired cognition are ≤9 p,[13, 15] ≤10 p,[16] and ≤6 p.[17, 28]

The SF-MoCA is based on items such as delayed recall, serial 7, and orientation.[11, 12] The total SF-MoCA score is 14 p, and a cutoff of ≤8 p has been suggested as an indicator for impaired cognition. [11, 12]

Statistics

Data will be presented as the mean and standard deviation, median and interquartile range, minimum–maximum (min–max), number (n), and proportion (%) (Supplementary Tables 2 and

For peer review only - http://bmjopen.bmj.com/site/about/guidelines.xhtml

3). The odds ratio and 95% confidence interval (CI) will be presented when appropriate. The significance level for the statistical tests will be set at $\alpha = 5\%$. All statistical tests will be two-tailed.

Objective 1

Two different approaches will be used to identify the MoCA items that have the potential to form the s-MoCA-SWE: exploratory factor analysis[29, 30] and boosted regression tree.[31]

Exploratory factor analysis is a dimension-reduction method with good sensitivity for identifying the latent connections between variables. Exploratory factor analysis will be performed in several steps. First, the correlations between the MoCA items will be calculated. Second, the results of Bartlett's and Kaiser–Meyer–Olkin tests will be evaluated; values lower than 0.05 and greater than 0.7, respectively, are desirable. Third, the number of factors will be determined based on the scree plot and parallel analysis scree plot of eigenvalues. A factor-extraction method will be chosen, and a decision about rotation will be made based on the results of the previous steps. MoCA items with loading >0.6 will be chosen as having the potential to enter the s-MoCA-SWE.[30]

Boosted regression tree is a supervised machine-learning algorithm. In the boosted regression tree algorithm, modelling trees are grown sequentially (e.g. each tree is grown based on the information from the previously grown trees and each tree is fit to the residuals from the previous tree). For this analysis, the dataset will be split into training (80%) and test (20%) datasets. The s-MoCA-SWE boosted regression tree will be developed based on the training dataset, and tuning parameters such as the number of trees (B), shrinkage parameter (λ), and interaction depths (d) will be chosen based on 10-fold cross-validation and bootstrapping. The MoCA items with the potential to build an s-MoCA-SWE will be chosen based on the boosted regression tree model with adjusted tuning parameters and variable coefficients >5.0. The s-MoCA-SWE developed based on the training dataset will be further tested on the test dataset.

The decision between the set of MoCA items derived from exploratory factor analysis and that derived from the boosted regression tree algorithm will be made by applying binary logistic regression analysis. The sets will be entered as independent variables and tested using different regression models. The dependent variable will be dichotomised MoCA (≤25 p for impaired cognition).[8, 32] The s-MoCA-SWE version with the highest sensitivity and classification accuracy for cognitive impairment will be analysed in Objective 2.

For peer review only - http://bmjopen.bmj.com/site/about/guidelines.xhtml

Objective 2

To find the optimal cutoff value of s-MoCA-SWE for classifying patients with and without cognitive impairment, binary logistic regression analysis will be performed. The MoCA cutoff of ≤25 p for impaired cognition will be the reference output,[8, 32] and the s-MoCA-SWE full score will be the independent variable. The cutoff value of s-MoCA-SWE will be chosen based on its good sensitivity for identifying patients with cognitive impairment.

Objective 3

Four cross tables will be evaluated. The reference variable will be dichotomised MoCA (≤25 p for impaired cognition).[8, 32] The index variables will be dichotomised s-MoCA-SWE (according to the results from Objective 2), T-MoCA (≤18 p for impaired cognition),[14] NINDS-CSN (≤9 p for impaired cognition),[13, 15] and SF-MoCA (≤8 p for impaired cognition).[11, 12] The psychometric measurements of sensitivity, specificity, positive predictive value, negative predictive value, and Youden's index with 95% CI will be evaluated.

Receiving operating characteristic curve analysis will be performed. The full scores of s-MoCA-SWE, T-MoCA, NINDS-CSN, and SF-MoCA will be entered as test variables, and the dichotomised MoCA (≤25 p for impaired cognition) will be entered as the state variable.[8, 32] The areas under the curve will be interpreted as follows: 0.7–0.9, moderate accuracy and 0.5–0.7, low accuracy.[33]

Subgroup analyses

The properties of the s-MoCA-SWE will be evaluated further. Subgroup analysis will be performed by studying the differences between patients with and without a previous stroke/TIA, with the hypothesis that patients with a previous stroke are more likely to have impaired cognition; by stroke severity, with the hypothesis that patients with more severe stroke are more likely to have more impaired cognition; and by age, with the hypothesis that older patients are more likely to have impaired cognition.

Missing data

For descriptive statistics, data on all included patients will be used. The proportion of missing values will be provided for each baseline variable.

For peer review only - http://bmjopen.bmj.com/site/about/guidelines.xhtml

Statistical software

Data will be analysed using SPSS Statistics for Windows version 27.0 (released on 2018; IBM Corp., Armonk, NY) and R version 4.0.2 (R Core Team. R: A language and environment for statistical computing. R Foundation for Statistical Computing, Vienna, Austria.).

Patient and public involvement statement

The patients and the public were not involved in the design of the protocol.

Cognition has been identified by patients, their caregivers, and healthcare professionals as one of the most important research areas in stroke. [34] Furthermore, many patients have reported unmet needs regarding problems with cognitive functions, such as memory, concentration, and speaking/reading. [35] Thus, early identification of cognitive impairment is important.

Patients with stroke can find cognitive assessments fairly burdensome in acute stroke and would prefer short tests if possible. By developing s-MoCA-SWE, we hope to have a simpler and more efficient cognitive screening tool that can be used during acute and subacute stroke. Theoretically, this would lead to a higher proportion of patients who receive the recommended cognitive screenings.

ETHICS AND DISSEMINATION

Väststroke: The study obtained ethics approval from the Regional Ethical Review Board in Gothenburg (346–16) and the Swedish Ethical Review Authority (amendment 2019–04299). According to the Swedish Data Protection Authority, the handling of data generated within the framework of quality registers represents an exception to the general rule of written informed consent being required from patients. Furthermore, the Personal Data Act (Swedish law #1998:204, issued 29 April 1998) allows data from medical charts to be collected for clinical purposes and quality control without written informed consent.

The EFFECTS study has received ethical approval from the Stockholm Ethics Committee (2013/1265-31/2 on 30/09/2013). All participants of the EFFECTS study provided written consent for inclusion in the randomised controlled trial. They also provided written and oral consent to merge their data with other registry data.

For peer review only - http://bmjopen.bmj.com/site/about/guidelines.xhtml

In order to disseminate the study findings to a wide audience, results will be published in international, peer-reviewed journals, presented at local and international conferences, communicated to clinical practitioners in local meetings and seminars, and shared via social media channels.[36] Furthermore, a short report will be published in Swedish journals that target professionals who perform clinical cognitive screenings.

COMPETING INTERESTS

None declared.

FUNDING

This work was supported by the IRIS Foundation, Swedish Research Council (VR2017-00946), Swedish Heart and Lung Foundation, Swedish Brain Foundation, Promobilia, Swedish state under an agreement between the Swedish government and the county councils, ALF agreement (ALFGBG-718711, ALFGBG-877961), Swedish National Stroke Association, Local Research and Development Board for Gothenburg and Södra Bohuslän, Greta and Einar Asker's Foundation, Rune and Ulla Almöv's Foundation for Neurological Research, Hjalmar Svensson's Research Foundation, P-O Ahl & J-B Wennerström's Foundation; Herbert & Karin Jacobssons Foundation, and Sahlgrenska University Hospital Foundations.

AUTHOR CONTRIBUTIONS

TA: conceptualisation of the study, statistics, drafting of the manuscript; EL: chief investigator of EFFECTS, providing of EFFECTS's data, critical revision of the manuscript; DB: statistical consultations, critical revision of the manuscript; ME: statistical consultations, critical revision of the manuscript; TQ: statistical consultations, conceptualisation of the study, critical revision of the manuscript; KSS: acquisition of Väststroke data, conceptualisation of the study, critical revision of the manuscript.

DATA AVAILABILITY STATEMENT

According to the Swedish regulations shown at https://etikprovning.se/for-forskare/ansvar/, the data cannot be publicly shared because of ethical and legal reasons. Researchers can request access to the data by emailing the principal investigators Katharina S. Sunnerhagen at

For peer review only - http://bmjopen.bmj.com/site/about/guidelines.xhtml

ks.sunnerhagen@neuro.gu.se (Väststroke) and Erik Lundström at erik.lundstrom@neuro.uu.se (EFFECTS).

References

1. McDonald MW, Black SE, Copland DA, et al. Cognition in stroke rehabilitation and recovery research: Consensus-based core recommendations from the second Stroke Recovery and Rehabilitation Roundtable. *Int J Stroke* 2019;**14**:774–782.
2. Sachdev PS, Blacker D, Blazer DG, et al. Classifying neurocognitive disorders: The DSM-5 approach. *Nat Rev Neurol* 2014;**10**:634–642.
3. Nys GMS, Van Zandvoort MJE, De Kort PLM et al. Cognitive disorders in acute stroke: Prevalence and clinical determinants. *Cerebrovasc Dis* 2007;**23**:408–416.
4. Abzhandadze T, Reinholdsson M, Sunnerhagen KS. NIHSS is not enough for cognitive screening in acute stroke: A cross-sectional, retrospective study. *Sci Rep* 2020;**10**: doi: 10.1038/s41598-019-57316-8.
5. Patel MD, Coshall C, Rudd AG et al. Cognitive impairment after stroke: Clinical determinants and its associations with long-term stroke outcomes. *J Am Geriatr Soc* 2002;**50**:700–706.
6. Eng JJ, Bird ML, Godecke E, et al. Moving stroke rehabilitation research evidence into clinical practice: Consensus-based core recommendations from the Stroke Recovery and Rehabilitation Roundtable. *Int J Stroke* 2019;**14**:766–773.
7. Quinn TJ, Elliott E, Langhorne P. Cognitive and mood assessment tools for use in stroke. *Stroke* 2018;**49**:483–490.
8. Nasreddine ZS, Phillips NA, Bédirian V, et al. The Montreal Cognitive Assessment, MoCA: A brief screening tool for mild cognitive impairment. *J Am Geriatr Soc* 2005;**53**:695–699.
9. Munthe-Kaas R, Aam S, Saltvedt I, et al. Test accuracy of the Montreal cognitive assessment in screening for early poststroke neurocognitive disorder: The nor-COAST study. *Stroke* 2021;**52**:317–320.
10. Chiti G, Pantoni L. Use of Montreal cognitive assessment in patients with stroke. *Stroke* 2014;**45**:3135–3140.
11. Horton DK, Hynan LS, Lacritz LH et al. An abbreviated Montreal cognitive assessment (MoCA) for dementia screening. *Clin Neuropsychol* 2015;**29**:413–425.
12. McDicken JA, Elliott E, Blayney G, et al. Accuracy of the short-form Montreal Cognitive Assessment: Systematic review and validation. *Int J Geriatr Psychiatry* 2019;**34**:1515–1525.
13. Pendlebury ST, Welch SJ, Cuthbertson FC et al. Telephone assessment of cognition after transient ischemic attack and stroke: Modified telephone interview of cognitive status and telephone Montreal Cognitive Assessment versus face-to-face Montreal Cognitive Assessment and neuropsychological battery. *Stroke* 2013;**44**:227–229.
14. Wittich W, Phillips N, Nasreddine ZS et al. Sensitivity and specificity of the Montreal cognitive assessment modified for individuals who are visually impaired. *J Vis Impairment Blindness* 2010;**104**:360–368.
15. Cameron JD, Gallagher R, Pressler SJ, et al. Sensitivity and specificity of a five-minute cognitive screening test in patients with heart failure. *J Card Fail* 2016;**22**:99–107.
16. Kaur D, Kumar G, Singh AK. Quick screening of cognitive function in Indian multiple sclerosis patients using Montreal cognitive assessment test-short version. *Ann Indian Acad Neurol* 2013;**16**:585–589.

For peer review only - http://bmjopen.bmj.com/site/about/guidelines.xhtml

17. Lim JS, Oh MS, Lee JH, et al. Prediction of post-stroke dementia using NINDS-CSN 5-minute neuropsychology protocol in acute stroke. *Int Psychogeriatr* 2017;**29**:777–784.
18. Lin HF, Chern CM, Chen HM, et al. Validation of NINDS-VCI neuropsychology protocols for vascular cognitive impairment in Taiwan. *PLoS One*. Public Library of Science 2016;11: doi:10.1371/journal.pone.0156404
19. Wei J, Jin X, Chen B, et al. Comparative study of two short-form versions of the Montreal cognitive assessment for screening of post-stroke cognitive impairment in a Chinese population. *Clin Interv Aging* 2020;**15**:907–914.
20. Wong A, Law LS, Liu W et al. Montreal cognitive assessment: One cutoff never fits all. *Stroke* 2015;**46**:3547–3550.
21. Stern Y. What is cognitive reserve? Theory and research application of the reserve concept. *J Int Neuropsychol Soc* 2002;**8**:448–460.
22. Shin M, Sohn MK, Lee J et al. Effect of cognitive reserve on risk of cognitive impairment and recovery after stroke: The KOSCO Study. *Stroke* 2020;**51**:99–107.
23. O'Driscoll C, Shaikh M. Cross-cultural applicability of the Montreal cognitive assessment (MoCA): A systematic review. *J Alzheimers Dis* 2017;**58**:789–801.
24. EFFECTS Trial Collaboration. Safety and efficacy of fluoxetine on functional recovery after acute stroke (EFFECTS): A randomised, double-blind, placebo-controlled trial. *Lancet Neurol* 2020;**19**:661–669.
25. Asplund K, Eriksson M, Riks-Stroke Collaboration. Inflammation, poststroke depression and statins. *Int J Stroke* 2011;**6**:567–568.
26. Bernhardt J, Hayward KS, Kwakkel G, et al. Agreed definitions and a shared vision for new standards in stroke recovery research: The Stroke Recovery and Rehabilitation Roundtable taskforce. *Int J Stroke* 2017;**12**:444–450.
27. Kasner SE, Chalela JA, Luciano JM, et al. Reliability and validity of estimating the NIH stroke scale score from medical records. *Stroke* 1999;**30**:1534–1537.
28. Dong Y, Xu J, Chan BP-L, et al. The Montreal Cognitive Assessment is superior to National Institute of Neurological Disease and Stroke-Canadian Stroke Network 5-minute protocol in predicting vascular cognitive impairment at 1 year. *BMC Neurol* 2016;**16**: doi: 10.1186/s12883-016-0570-y.
29. Child D. Essentials of factor analysis. 3. *Educ Educ*. New York: NY: Continuum 2006.
30. Kline P. An easy guide to factor analysis. *London: london*. Routledge 1994.
31. Chen T, Guestrin C. *XGBoost: A scalable tree boosting system. Ithaca2016*.
32. Borland E, Nägga K, Nilsson PM et al. The Montreal cognitive assessment: Normative data from a Large Swedish population-based cohort. *J Alzheimers Dis* 2017;**59**:893–901.
33. Fischer JE, Bachmann LM, Jaeschke R. A readers' guide to the interpretation of diagnostic test properties: Clinical example of sepsis. *Intensive Care Med* 2003;**29**:1043–1051.
34. Pollock A, St George B, Fenton M et al. Top 10 research priorities relating to life after stroke - Consensus from stroke survivors, caregivers, and health professionals. *Int J Stroke* 2014;**9**:313–320.
35. McKevitt C, Fudge N, Redfern J, et al. Self-reported long-term needs after stroke. *Stroke* 2011;**42**:1398–1403.
36. Edwards DJ. Dissemination of research results: On the path to practice change. *Can J Hosp Pharm* 2015;**68**:465–469.

For peer review only - http://bmjopen.bmj.com/site/about/guidelines.xhtml

Supplementary File

Development of a short-form Swedish version of the Montreal Cognitive Assessment (s-MoCA-SWE): Protocol for a cross-sectional study

Tamar Abzhandadze[1,2], Erik Lundström[3], Dongni Buvarp[1], Marie Eriksson[4], Terence J. Quinn [5], Katharina S. Sunnerhagen[1,6].

[1]Institute of Neuroscience and Physiology, The Sahlgrenska Academy, University of Gothenburg, Gothenburg, Sweden.

[2]Department of Occupational Therapy and Physiotherapy, Sahlgrenska University Hospital, Gothenburg, Sweden.

[3]Department of Neuroscience, Neurology, Uppsala University, Akademiska Sjukhuset, Uppsala, Sweden.

[4]Department of Statistics, USBE, Umeå University, Umeå, Sweden.

[5]Institute of Cardiovascular and Medical Sciences, University of Glasgow, Glasgow, UK.

[6] Neurocare, Sahlgrenska University Hospital, Gothenburg, Sweden.

Correspondence to:

Tamar Abzhandadze

Institute of Neuroscience and Physiology

Rehabilitation Medicine, University of Gothenburg

Per Dubbsgatan 14, fl. 3

413 45 Gothenburg, Sweden

E-mail: tamar.abzhandadze@gu.se, tamar.abzhandadze@vgregion.se

For peer review only

Supplementary Table 1. Description of variables and their categories respective data set and in the merged dataset.

Väststroke	Väststroke: variable categories and coding	EFFECTS dataset	EFFECTS: variable categories and coding	Merged dataset: variable names and coding
				Cohorts, binary:<br>1 = Väststroke<br>2 = EFFECTS
Sex	Binary:<br>0 = female<br>1 = male	Pat_Gender	F = female<br>M = male	Sex, binary:<br>1 = female<br>2 = male
Age	Scale: range	AGE	Numeric	Age: numeric
Living arrangement/living alone prior to stroke	Binary:<br>1 = no<br>2 = yes	pat_Living_Arrangements	A = living alone<br>B = institutional living<br>C = other living arrangement<br>D = living with someone else	Living arrangement prior to stroke, binary:<br>1 = lived alone (Väststroke = 2, EFFECTS = A)<br>2 = did not live alone (Väststroke = 1, EFFECTS ≥B)
Needing help/dependent prior to stroke	Binary:<br>1 = no<br>2 = yes	Stroke daily	N = no<br>Y = yes<br><br>*Note: "N" means that the patient did not require assistance with ADLs prior to stroke.*	Needs assistance with ADL before stroke, binary:<br>1 = no<br>2 = yes
Diabetes	Binary:<br>0 = no<br>1 = yes	DIABETES	N = no<br>Y = yes<br>U = unknown	Diabetes, nominal:<br>0 = no<br>1 = yes<br>2 = unknown

For peer review only

TIA prior to stroke	Binary:<br>0 = no<br>1 = yes	PREV_Stroke_TIA<br><br>*Note: In EFFECTS, previous TIA and previous ischemic stroke are not distinguished.*	N = no<br>Y = yes<br>U = unknown	Previous TIA or ischemic stroke, nominal:<br>0 = no<br>1 = yes<br>2 = unknown
Stroke diagnosis	Nominal, 3 levels:<br>1 = I61<br>2 = I63<br>3 = I64	Diagnosis	1 = ischemic stroke<br>2 = intracerebral haemorrhage<br>3 = non-stroke	Stroke diagnosis, nominal:<br>1 = I63<br>2 = I61<br>3 = I64
Reperfusion	1 = both IVT and endovascular thrombectomy<br>2 = endovascular thrombectomy only<br>3 = IVT only	Thrombolysis_Trombectomy	1 = both IVT and endovascular thrombectomy<br>2 = endovascular thrombectomy only<br>3 = IVT only	Reperfusion treatment, nominal:<br>1 = both IVT and endovascular thrombectomy<br>2 = endovascular thrombectomy only<br>3 = IVT only
NIHSS awareness	Ordinal: range 0–3	Stroke_NIHSS_1a	0 = alert<br>1 = not alert, but arousable with minimal stimulation<br>2 = not alert, requires repeated stimulation<br>3 = coma	NIHSS 1a, awareness, ordinal:<br>0 = alert<br>1 = not alert, but arousable with minimal stimulation<br>2 = not alert, requires repeated stimulation<br>3 = coma
NIHSS orientation	Ordinal: range 0–2	Stroke_NIHSS_1b	0 = answers both correctly<br>1 = answers one correctly<br>2 = answers both incorrectly	NIHSS 1b, orientation, ordinal:<br>0 = answers both correctly<br>1 = answers one correctly<br>2 = answers both incorrectly

For peer review only

NIHSS understanding	Ordinal: range 0–2	Stroke_NIHSS_1c	0 = obeys both correctly<br>1 = obeys one correctly<br>2 = obeys both incorrectly	NIHSS 1c, understanding, ordinal:<br>0 = obeys both correctly<br>1 = obeys one correctly<br>2 = obeys both incorrectly
NIHSS eye movement	Ordinal: range 0–2	Stroke_NIHSS_2	0 = normal<br>1 = partial gaze palsy<br>2 = total gaze paresis	NIHSS 2, eye movement, ordinal:<br>0 = normal<br>1 = partial gaze palsy<br>2 = total gaze paresis
NIHSS visual field	Ordinal: range 0–3	Stroke_NIHSS_3	0 = no visual field loss<br>1 = partial hemianopia<br>2 = complete hemianopia<br>3 =bilateral hemianopia	NIHSS 3, visual field, ordinal:<br>0 = no visual field loss<br>1 = partial hemianopia<br>2 = complete hemianopia<br>3 = bilateral hemianopia
NIHSS facial palsy	Ordinal: range 0–3	Stroke_NIHSS_4	0 = normal symmetrical movement<br>1 = minor paralysis<br>2 = partial paralysis<br>3 = complete paralysis	NIHSS 4, facial palsy, ordinal:<br>0 = normal symmetrical movement<br>1 = minor paralysis<br>2 = partial paralysis<br>3 = complete paralysis
NIHSS motor arm: right	Ordinal: range 0–4	Stroke_NIHSS_7	0 = normal<br>1 = drift<br>2 = some effort against gravity<br>3 = no effort against gravity<br>4 = no effort against gravity	NIHSS 5, right motor arm, ordinal:<br>0 = normal<br>1 = drift<br>2 = some effort against gravity<br>3 = no effort against gravity<br>4 = no effort against gravity

For peer review only

NIHSS motor arm: left	Ordinal: range 0–4	Stroke_NIHSS_8	0 = normal<br>1 = drift<br>2 = some effort against gravity<br>3 = no effort against gravity<br>4 = no effort against gravity	NIHSS 6, left motor arm, ordinal:<br>0 = normal<br>1 = drift<br>2 = some effort against gravity<br>3 = no effort against gravity<br>4 = no effort against gravity
NIHSS motor leg: right	Ordinal: range 0–4	Stroke_NIHSS_5	0 = normal<br>1 = drift<br>2 = some effort against gravity<br>3 = no effort against gravity<br>4 = no effort against gravity	NIHSS 7, right motor leg, ordinal:<br>0 = normal<br>1 = drift<br>2 = some effort against gravity<br>3 = no effort against gravity<br>4 = no effort against gravity
NIHSS motor leg: left	Ordinal: range 0–4	Stroke_NIHSS_6	0 = normal<br>1 = drift<br>2 = some effort against gravity<br>3 = no effort against gravity<br>4 = no effort against gravity	NIHSS 8, left motor leg, ordinal:<br>0 = normal<br>1 = drift<br>2 = some effort against gravity<br>3 = no effort against gravity<br>4 = no effort against gravity
NIHSS ataxia	Ordinal: range 0–2	Stroke_NIHSS_9	0 = no ataxia<br>1 = present in one limb<br>2 = present in two limbs	NIHSS 9, ataxia, ordinal:<br>0 = no ataxia<br>1 = present in one limb<br>2 = present in two limbs
NIHSS sensory	Ordinal: range 0–2	Stroke_NIHSS_10	0 = normal<br>1 = mild-to-moderate sensory loss<br>2 = severe or total sensory loss	NIHSS 10, sensory, ordinal:<br>0 = normal<br>1 = mild-to-moderate sensory loss<br>2 = severe or total sensory loss

For peer review only

NIHSS language	Ordinal: range 0–3	Stroke_NIHSS_11	0 = no aphasia<br>1 = mild-to-moderate aphasia<br>2 = severe aphasia<br>3 = mute	NIHSS 11, language, ordinal:<br>0 = no aphasia<br>1 = mild-to-moderate aphasia<br>2 = severe aphasia<br>3 = mute
NIHSS dysarthria	Ordinal: range 0–2	Stroke_NIHSS_12	0 = normal<br>1 = mild-to-moderate dysarthria<br>2 = severe dysarthria<br>3 = untestable	NIHSS 12, dysarthria, ordinal:<br>0 = normal<br>1 = mild-to-moderate dysarthria<br>2 = severe dysarthria<br>3 = untestable
NIHSS neglect	Ordinal: range 0–2	Stroke_NIHSS_13	0 = normal<br>1 = inattention to one modality<br>2 = severe hemi-inattention	NIHSS 13, neglect, ordinal:<br>0 = normal<br>1 = inattention to one modality<br>2 = severe hemi-inattention
NIHSS total score	Ordinal: range 0–36	NIHSS	Ordinal: range 0–26	NIHSS total score, ordinal: range 0–36
MoCA visuospatial/executive functions: 1A + cube + clock	Ordinal: range 0–5	MOCA_Base_Viso_exe	Ordinal: range 0–5	MoCA visuospatial/executive functions: trial + cube + clock, ordinal: range 0–5
MoCA task: naming	Ordinal: range 0–3	MOCA_Base_Name	Ordinal: range 0–3	MoCA: naming, ordinal: range 0–3
MoCA attention task: numbers forward and backward	Ordinal: range 0–2	MOCA_Base_Att_fwd_back	Ordinal: range 0–2	MoCA: digit span, ordinal: range 0–2
MoCA attention task: knock on A	Nominal:<br>0 = no point<br>1 = 1 point	MOCA_Base_Att_knock	Nominal:<br>0 = no point<br>1 = 1 point	MoCA: tap on A, binary:<br>0 = no point<br>1 = 1 point
MoCA attention task: subtraction 100 - 7	Ordinal: range 0–3	MOCA_Base_Att_subtract	Ordinal: range 0–3	MoCA: serial 7, ordinal: range 0–3

For peer review only

MoCA language task: sentence repetition, 2	Ordinal: range 0–2	MOCA_Base_Lang_rep	Ordinal: range 0–2	MoCA: repetition, ordinal: range 0–2
MoCA language task: fluency, F word	Nominal:<br>0 = no point<br>1 = 1 point	MOCA_Base_Fluency	Nominal:<br>0 = no point<br>1 = 1 point	MoCA: fluency, binary:<br>0 = no point<br>1 = 1 point
MoCA abstraction task: categorizing of two tasks	Ordinal: range 0–2	MOCA_Base_Abstraction	Ordinal: range 0–2	MoCA: abstraction, ordinal: range 0–2
MoCA memory task: recall of five words	Ordinal: range 0–5	MOCA_Base_Delay	Ordinal: range 0–5	MoCA: delayed recall, ordinal: range 0–5
MoCA orientation task: six questions	Ordinal: range 0–6	MOCA_Base_Orientation	Ordinal: range 0–6	MoCA: orientation, ordinal: range 0–6
MoCA total score	Ordinal: range 1–30	MOCA_Base_Total	Ordinal: range 1–30	MoCA total score, ordinal: range 1–30
MoCA school ≤12 years, +1 point	Binary:<br>0 = no<br>1 = yes, +1 point	MOCA_Education	0 = no<br>1 = yes, +1 point	MoCA education, binary:<br>0 = no,<br>1 = yes, +1 point
				A telephone version of the MoCA, ordinal: range 0–22<br><br>Aggregated score of the MoCA items: delayed recall, digit span, list letters, serial 7, sentence repetition, fluency, abstraction, and orientation
				Short-form MoCA, ordinal: range 0–14<br><br>Aggregated score of the MoCA items: delayed recall, serial 7, and orientation

For peer review only

				NINDS-CSN–MoCA, ordinal: range 0–12<br><br>Aggregated score of the MoCA items: delayed recall, fluency, and orientation
Discharge destination*	Ordinal, 10 levels:<br>1 = own accommodation<br>2 = arranged accommodation<br>4 = another acute-care department<br>5 = geriatrics/rehabilitation<br>6 = deceased during treatment<br>7 = other acute-care department (e.g. the patient is resident in another country)<br>9 = not known<br>11 = still in hospital<br>12 = another stroke unit for aftercare<br>13 = health centre with emergency beds	Discharge residence	1 = own home<br>2 = relatives' home<br>3 = residential home<br>4 = long-term NHS home<br>6 = another hospital<br>7 = other	Discharge destination, ordinal:<br>1 = own or relatives' home (Väststroke 1, EFFECTS 1 and 2)<br>2 = residential home (Väststroke 2, EFFECTS 3 and 4)<br>3 = other hospital (Väststroke 4, 5, 11, and 12, EFFECTS 6)<br>4 = other (Väststroke 7, 9, and 13, EFFECTS 7)

Abbreviations: ADL, activity of daily living; IVT, intravenous thrombolysis; MoCA, Montreal Cognitive Assessment; NIHSS, National Institutes of Health Stroke Scale; NINDS-CSN, National Institute of Neurological Disorders and Stroke–Canadian Stroke Network; TIA, transient ischemic attack.

For peer review only

Supplementary Table 2. Patient baseline characteristics (template table)

	Categories	Total sample<br>N =	Väststroke<br>N =	EFFECTS<br>N =
Sex, n (%)	Male<br>Female			
Age, y, mean (SD)				
Living arrangement, n (%)	Lives alone<br>Living with someone else			
Education, 1 point for a maximum of 12 years, n (%)	Yes<br>No			
Assistance with ADLs prior to stroke, n (%)	No<br>Yes			
Having diabetes, n (%)	No<br>Yes<br>Unknown			
Previous TIA or ischemic stroke, n (%)	No<br>Yes<br>Unknown			
Stroke diagnosis, n (%)	I61<br>I63<br>I64			

For peer review only

Reperfusion treatment, n (%)	Both IVT and ET<br>ET only<br>IVT only			
NIHSS, median (min–max)				
MoCA, median (min–max)				
Discharge destination, n (%)	Own or relatives' home<br>Residential or long-term<br>NHS home<br>Other hospitals<br>Other			

Note: The sum may vary due to missing data.

Abbreviations: ADL, activity of daily living; IVT, intravenous thrombolysis; IQR, interquartile range; MoCA, Montreal Cognitive Assessment; NIHSS, National Institutes of Health Stroke Scale; NHS, National Health System; SD, standard deviation; ET, endovascular thrombectomy; TIA, transient ischemic attack.

Variables with missing values, n (%):

For peer review only

Supplementary Table 3. Description of cognitive functions (template table).

		Total sample<br>N =	Väststroke<br>N =	EFFECTS<br>N =
	Points	n (%)	n (%)	n (%)
Visuospatial/executive functions	0<br>1<br>2<br>3<br>4<br>5			
Naming three animals	0<br>1<br>2<br>3			
Digit span	0<br>1<br>2			
Tap on A	0<br>1			
Serial 7	0<br>1			

For peer review only

	2<br>3			
Repetition	0<br>1<br>2			
Fluency	0<br>1			
Abstraction	0<br>1<br>2			
Delayed recall	0<br>1<br>2<br>3<br>4<br>5			
Orientation	0<br>1<br>2<br>3<br>4			

For peer review only

	5<br>6			
MoCA score	Median (IQR)			
T-MoCA	Median (IQR)			
NINDS-CSN–MoCA	Median (IQR)			
SF-MoCA	Median (IQR)			

Abbreviations: IQR, interquartile range; MoCA, Montreal Cognitive Assessment (score range: 1–30); NINDS-CSN–MoCA, National Institute of Neurological Disorders and Stroke–Canadian Stroke Network MoCA (score range: 0–12); SF-MoCA, short-form MoCA (score range: 0–14); T-MoCA, a telephone version of the MoCA (score range: 0–22).

For peer review only

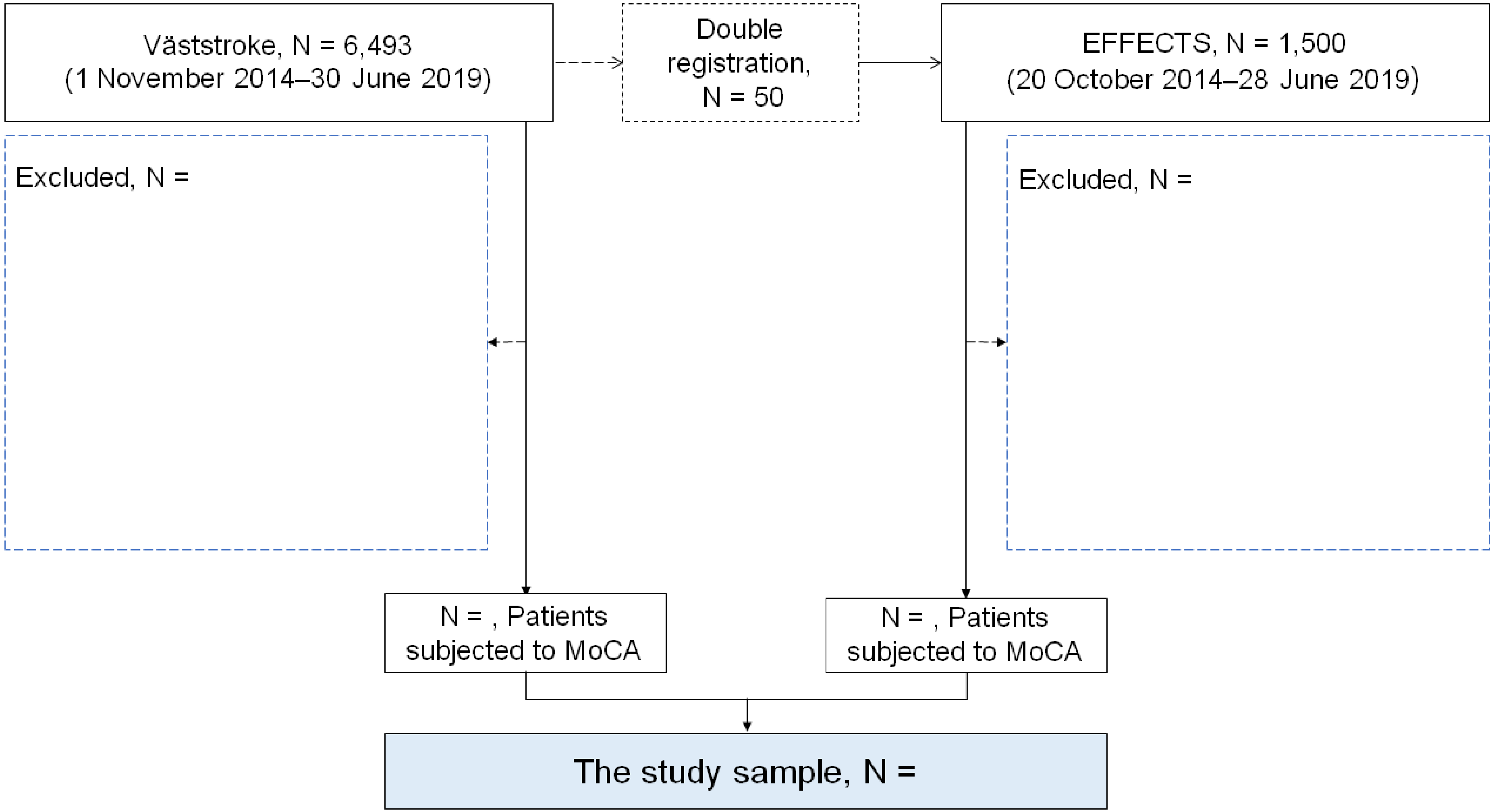

Supplementary Figure. Flowchart of patient selection (template diagram). Abbreviations: MoCA, the Montreal Cognitive Assessment; EFFECTS, **E**fficacy o**F** **F**luoxetine—a randomis**E**d **C**ontrolled **T**rial in **S**troke.
